# A near‐complete genome assembly of *Brassica rapa* provides new insights into the evolution of centromeres

**DOI:** 10.1111/pbi.14015

**Published:** 2023-02-02

**Authors:** Lei Zhang, Jianli Liang, Haixu Chen, Zhicheng Zhang, Jian Wu, Xiaowu Wang

**Affiliations:** ^1^ State Key Laboratory of Vegetable Biobreeding, Institute of Vegetables and Flowers Chinese Academy of Agricultural Sciences Beijing China

**Keywords:** *Brassica rapa*, near‐complete genome assembly, LTRs, centromeres, evolution

## Abstract

*Brassica rapa* comprises many important cultivated vegetables and oil crops. However, Chiifu v3.0, the current *B. rapa* reference genome, still contains hundreds of gaps. Here, we presented a near‐complete genome assembly of *B. rapa* Chiifu v4.0, which was 424.59 Mb with only two gaps, using Oxford Nanopore Technology (ONT) ultralong‐read sequencing and Hi‐C technologies. The new assembly contains 12 contigs, with a contig N50 of 38.26 Mb. Eight of the ten chromosomes were entirely reconstructed in a single contig from telomere to telomere. We found that the centromeres were mainly invaded by *ALE* and *CRM* long terminal repeats (LTRs). Moreover, there is a high divergence of centromere length and sequence among *B. rapa* genomes. We further found that centromeres are enriched for *Copia* invaded at 0.14 MYA on average, while pericentromeres are enriched for *Gypsy* LTRs invaded at 0.51 MYA on average. These results indicated the different invasion mechanisms of LTRs between the two structures. In addition, a novel repetitive sequence *PCR630* was identified in the pericentromeres of *B. rapa*. Overall, the near‐complete genome assembly, *B. rapa* Chiifu v4.0, offers valuable tools for genomic and genetic studies of *Brassica* species and provides new insights into the evolution of centromeres.

## Introduction

Decoding complete genome sequence information is vital for understanding genome structure and further facilitating the genetic improvement of critical agronomic traits. Recent advances in long‐read sequencing technologies, such as Pacific Biosciences (PacBio) and Oxford Nanopore Technology (ONT), have led to a paradigm shift in our ability to obtain chromosome sequences from telomere to telomere. Recently, the Telomere‐to‐Telomere (T2T) consortium proposed a complete sequence of a human genome using PacBio HiFi and ONT ultralong‐read sequencing (Nurk *et al*., 2022). In plants, telomere‐to‐telomere and gapless genomes were presented using PacBio HiFi or ONT reads for *Arabidopsis thaliana* (Hou *et al*., 2022; Naish *et al*., 2021; Wang *et al*., 2021), rice (Li *et al*., 2021b; Song *et al*., 2021; Zhang *et al*., 2022b), and watermelon (Deng *et al*., 2022).

The *Brassica* genus encompasses many vegetables and oil crops. Six *Brassica* species, comprising three diploid species, *Brassica rapa* (A genome), *Brassica nigra* (B genome), and *Brassica oleracea* (C genome), as well as the three amphidiploid species, *Brassica juncea* (A and B genomes), *Brassica napus* (A and C genomes), and *Brassica carinata* (B and C genomes), form the famous “triangle of U” (Nagaharu, 1935). *Brassica* species not only shared the whole genome duplication event at ~13–17 million years ago (MYA) with their close relative *A. thaliana* but also underwent the *Brassica*‐specific whole‐genome triplication event at ~5–9 MYA (Wang *et al*., 2011b). *B. rapa* formed many different subspecies with highly diverse morphotypes, such as heading Chinese cabbage, non‐heading pak choi, enlarged turnip tuber and oil seed crop yellow sarson (Cheng *et al*., 2016).

Using Chiifu‐401‐42 (Chinese cabbage), we achieved the first reference genome, Chiifu v1.5, among the *Brassica* species (Wang *et al*., 2011b). With the development of sequencing technologies, two updated versions of genome assemblies (Chiifu v2.5 and v3.0) and one updated version of genome annotation (Chiifu v3.5) were generated for *B. rapa* (Cai *et al*., 2017; Zhang *et al*., 2018, 2022c). Among these versions, Chiifu v1.5 and v2.5 were assembled with Illumina short reads (Cai *et al*., 2017; Wang *et al*., 2011b). Chiifu v3.0 was generated using a combination of PacBio, optical maps and chromosome conformation capture (Hi‐C) technologies (Zhang *et al*., 2018). Chiifu v3.5 is the updated annotation of Chiifu v3.0 using full‐length PacBio RNA sequencing technology (Zhang *et al*., 2022c). Recently, other *B. rapa* morphotypes have been sequenced using PacBio or ONT technologies, such as Chinese cabbage (Sun *et al*., 2022), pak choi (Li *et al*., 2020, 2021a,c; Xu *et al*., 2022), turnip (Park *et al*., 2021; Yang *et al*., 2022) and yellow sarson (Istace *et al*., 2021). In addition, a pan‐genome of *B. rapa* was released, including 16 genomes assembled using Illumina and PacBio reads (Cai *et al*., 2021). These *B. rapa* genome assemblies essentially promoted comparative genomics and genetic breeding studies of *Brassica* species. However, missing sequences and hundreds of gaps remain in these released genomes.

Centromeres are constricted regions of chromosomes responsible for attaching chromosomes to spindle microtubules during cell division (Comai *et al*., 2017). Centromeres and flanking pericentromeres comprise repetitive DNA sequences, such as long terminal repeats (LTRs) and satellite DNA (Talbert and Henikoff, 2022). In addition, centromeres contain many active genes (Nagaki *et al*., 2004). The completeness of centromeres in model plants *Arabidopsis* and rice has offered an intriguing understanding of their structure and function, including the enrichment of satellite DNA (Naish *et al*., 2021) and high methylation levels (Song *et al*., 2021). Recent studies have also shown that centromeres are highly variable in terms of organization and sequences, even among very closely related species, reflecting the rapid evolution of centromeres (Talbert and Henikoff, 2022). It was reported that *ATHILA* LTRs invade *Arabidopsis* centromeres CEN4 and CEN5 (Naish *et al*., 2021), and young *ALE* LTRs are predominantly amplified in the centromeres of *B. nigra* (Perumal *et al*., 2020). The centromere‐specific repeats (Cent‐SRs), including *CentBr1*, *CentBr2, CRB* and *TR805* (Koo *et al*., 2011; Lim *et al*., 2005, 2007), were widely used to determine the centromere boundary in *B. rapa* (Li *et al*., 2021a,b,c; Sun *et al*., 2022; Yang *et al*., 2022; Zhang *et al*., 2018), *B. nigra* (*CRB*, Perumal *et al*., 2020), *B. oleracea* (Cai *et al*., 2020; Guo *et al*., 2021), *B. juncea* (Kang *et al*., 2021) and *B. napus* (Rousseau‐Gueutin *et al*., 2020). Based on bacterial artificial chromosome clones, Lim *et al*. (2007) characterized the pericentromere‐specific repeats (Peri‐SRs) of *B. rapa*, including *PCRBr* and *TR238*. Due to the poor assembly of the repetitive regions in the previously published *Brassica* reference genomes, a global view of the size, structure and evolution of centromeres and pericentromeres remains elusive.

In this study, we presented a near‐complete genome assembly of *B. rapa* using a combination of ONT ultralong‐read sequencing and Hi‐C technologies. Our new assembly, *B. rapa* Chiifu v4.0, achieves the highest continuity and completeness among the published *B. rapa* genomes, which provides the opportunity for global analysis of centromeres and pericentromeres. Centromeres were mainly invaded by *ALE* and *CRM* LTRs and showed high divergence among different *B. rapa* genomes. Moreover, the LTRs in pericentromeres are much older than those in centromeres of *B. rapa*.

## Results

### A near‐complete *B. rapa* genome assembly

To achieve a high‐quality *B. rapa* genome assembly, Chinese cabbage (Chiifu‐401‐42) was sequenced using ONT technologies. In total, 90.24 Gb (~180 × coverage) of ONT reads were generated from the Promethion platform. We assembled ONT long reads using NextDenovo (v2.5, https://github.com/Nextomics/NextDenovo) and polished the resulting contigs with corrected ONT reads and Illumina reads. We filled the gaps with corrected ONT long reads and generated 12 contigs with a contig N50 of 38.26 Mb (Table 1). After scaffolding using our previous Hi‐C data (Zhang *et al*., 2018), we anchored all contigs onto ten chromosomes. Our final genome assembly, termed *B. rapa* Chiifu v4.0, had 424.59 Mb sequences with only two gaps on chromosomes A05 and A08, while the other eight chromosomes were reconstructed in a single contig from telomere to telomere (Table 1).

**Table 1:** Summary of *Brassica rapa* genome assemblies

Item	Chinese cabbage	Chinese cabbage	Chinese cabbage	Pak choi	Pak choi	Pak choi	Turnip	Yellow sarson
	Chiifu v4.0 (This study)	Chiifu v3.0 (Zhang *et al*., 2018)	assembly “A03” (Sun *et al*., 2022)	PC‐fu (Xu *et al*., 2022)	NHCC001 (Li *et al*., 2020)	ZYCX (Li *et al*., 2021c)	ECD04 (Yang *et al*., 2022)	Z1 v2 (Istace *et al*., 2021)
Estimated genome size (Mb)	455	455	455	478	478	478	518	529
Assembly size (Mb)	424.59	353.14	403.20	411.40	405.33	370.42	350.34	443.95
Contig number	12	1498	1222	2288	602	1985	1275	299
Contig N50 (kb)	38 257	1446	4290	4700	2830	2820	1520	10 256
Gap‐free chromosome number	8	None	None	None	None	None	None	None
Gaps number	2	407	1160	986	291	993	1203	85
Gene models	47 531	46 250	47 779	52 511	48 158	45 363	48 094	56 073
GC Content (%)	37.59	36.83	36.83	37.68	37.13	37.12	36.78	37.20
TE proportion (%)	53.78	45.84	50.99	63.30	46.15	39.80	42.71	52.97
Completeness (% BUSCO)	99.40	97.70	98.60	99.20	99.07	98.10	97.50	96.30
LTR assembly index score	15.05	9.69	7.61	3.99	11.19	8.11	12.19	5.64

**Figure 1:**
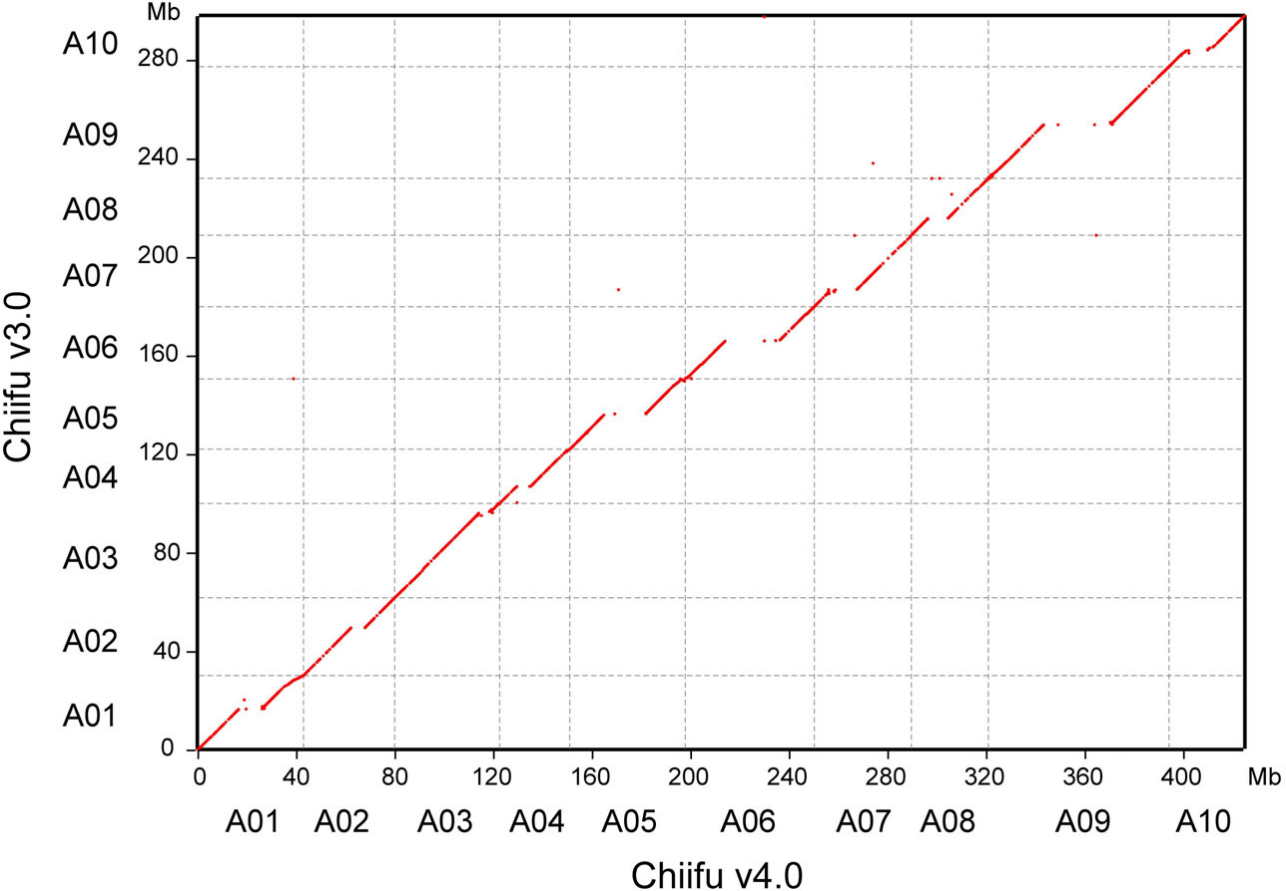
Chromosome collinearity between *Brassica rapa* Chiifu v4.0 and v3.0. The collinear regions between Chiifu v4.0 and v3.0 are shown in red dots. Chromosomes of Chiifu v4.0 are shown on the *x*‐axis, and chromosomes of Chiifu v3.0 are shown on the *y*‐axis.

The accuracy and completeness of Chiifu v4.0 were validated using multiple methods. First, the Hi‐C heatmap shows high consistency across all chromosomes, demonstrating the correct ordering and orientation of contigs in the new assembly (Figure S1). Second, the new assembly has high collinearity with Chiifu v3.0 (Figure 1). Third, genome accuracy was demonstrated by the high mapping rates of two raw sequences on the new assembly, including 99.73% (1 657 704/1 662 217) of ONT reads and 100% (1083/1083) of BAC sequences mapped on the new assembly. The sequence error after correction was 0.46%, estimated by Qualimap (v.2.2.1; Okonechnikov *et al*., 2016). Finally, for gene content assessment, our assembly captured 99.40% of the BUSCO 1614 reference gene set (Simao *et al*., 2015; Table 1). In addition, misassembled regions (371 kb) of Chiifu v3.0 were corrected in this new assembly, which were further validated by the Hi‐C heatmap and ONT reads (Figure S2).

We employed MAKER‐P (Campbell *et al*., 2014) to annotate the new assembly with the same evidence used to annotate Chiifu v3.0 (Zhang *et al*., 2018). To maintain consistency across different Chiifu versions, 47 233 protein‐coding genes (99.97%) were lifted from Chiifu v3.5 (47 249; Zhang *et al*., 2022c). Combining 298 gene models annotated with MAKER‐P in the newly assembled regions, the final annotation of the new assembly contained 47 531 gene models. We used EDTA (Ou *et al*., 2019) to annotate the repetitive sequences in the new assembly. In total, 393 202 transposable elements (TEs) were identified in the new assembly, accounting for ~53.78% (228.35 Mb/424.59 Mb) of Chiifu v4.0, approximately 10% greater than that of Chiifu v3.0 (Table 1).

**Figure 2:**
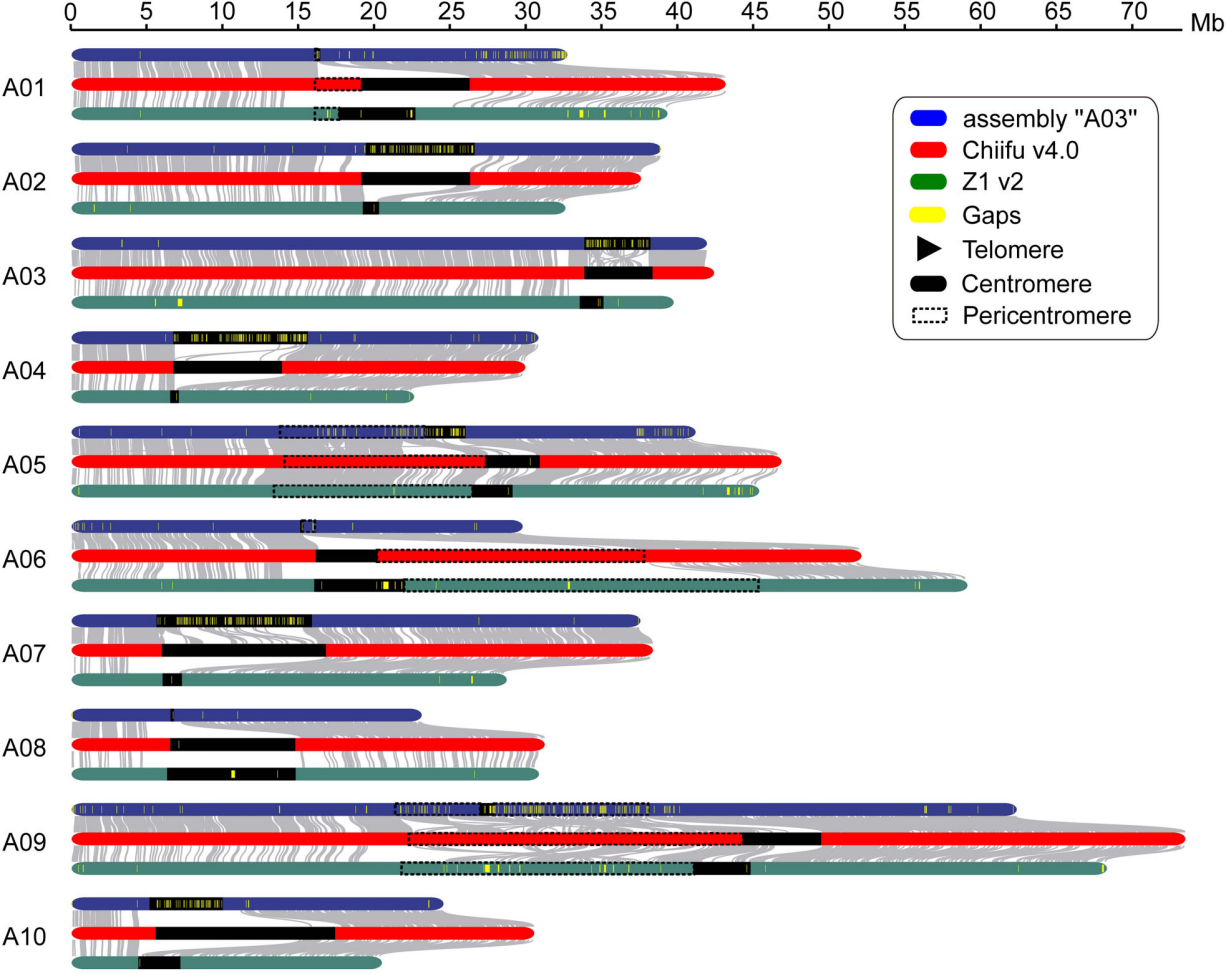
Chromosome collinearity between *Brassica rapa* Chiifu v4.0, assembly “A03” and Z1 v2. Grey lines link the collinear regions, and yellow blocks show the gaps. Black blocks indicate centromeres, and dashed blocks indicate pericentromeres.

Compared with Chiifu v3.0, we added ~71.45 Mb of novel sequences in the near‐complete assembly, almost all of which (98.64%, 70.48 Mb/71.45 Mb) were in the centromeres and pericentromeres (Figure S3). Moreover, all two gaps of Chiifu v4.0 were in the centromeric regions (Figure S4). We further compared Chiifu v4.0 with the other *B. rapa* genome assemblies based on long‐read sequencing, including a Chinese cabbage (assembly “A03”; Sun *et al*., 2022), three pak choi (PC‐fu, Xu *et al*., 2022; NHCC001, Li *et al*., 2020; ZYCX, Li *et al*., 2021c), a turnip (ECD04, Yang *et al*., 2022) and a yellow sarson (Z1 v2, Istace *et al*., 2021). Among them, Chiifu v4.0 had not only the longest contig N50 (38.26 Mb) and the fewest gaps (2) but also the highest values of the BUSCO (99.40%) and LTR assembly index (LAI) score (15.05) (Figures 2 and S5; Table 1). These results suggested that Chiifu v4.0 achieved the highest continuity and completeness among *B. rapa* genome assemblies.

### Invasion of *B. rapa* centromeres by *ALE* and *CRM* LTRs

**Figure 3:**
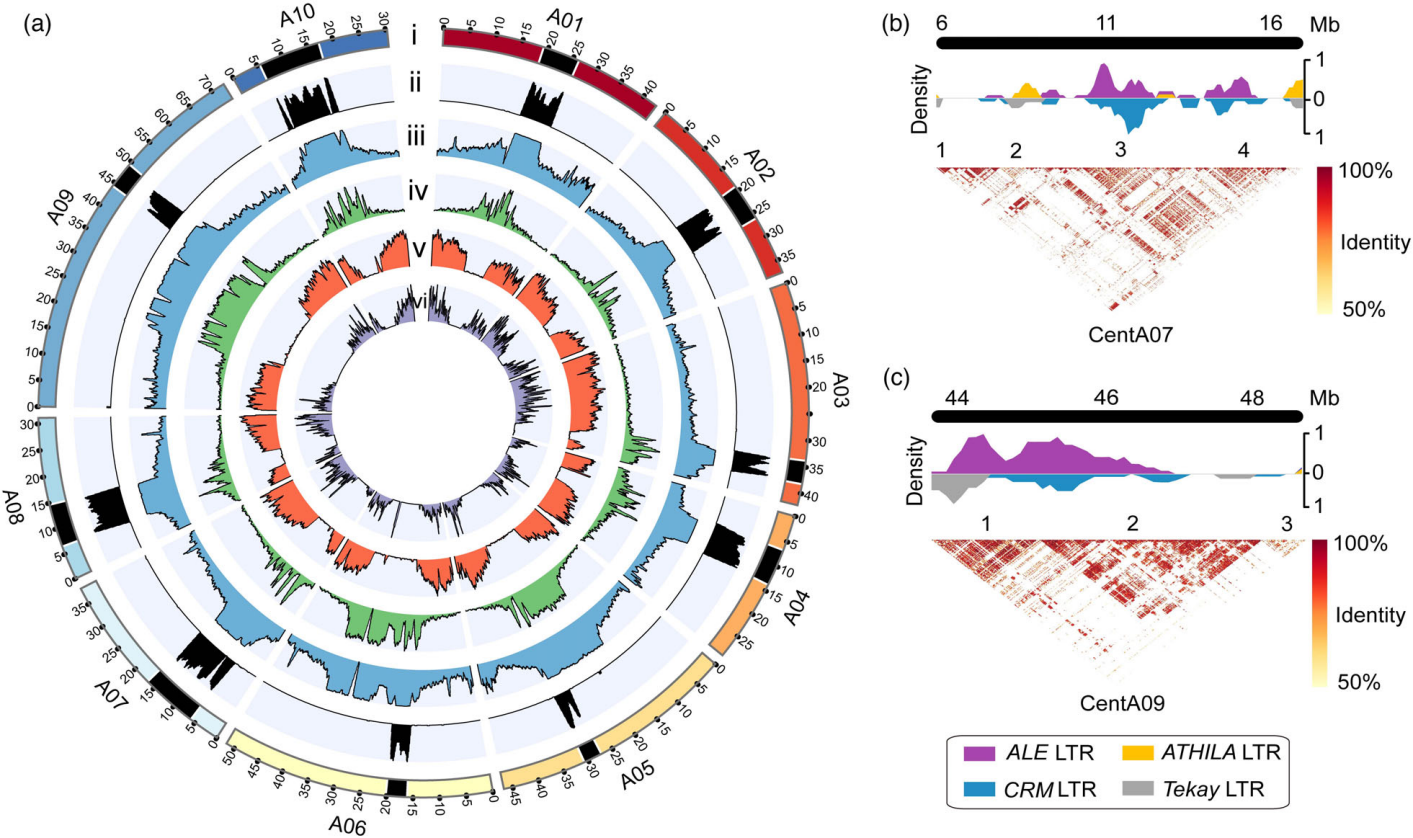
Characterization of the centromeres in *Brassica rapa* Chiifu v4.0. (a) Circos plot of Chiifu v4.0. i: Ten chromosomes of Chiifu v4.0. Centromeres are shown as black blocks. ii: Distribution of centromere‐specific repeats along chromosomes. iii: TE density across the chromosomes. iv: LTRs density across the chromosomes. v: Gene density of the chromosomes. vi: Expression level of genes along the chromosomes. (b,c) LTR distribution and heatmap of sequence identity in centromeres CentA07 (b) and CentA09 (c). Heatmap shows pairwise sequence identity between all non‐overlapping 10 kb regions of centromere. Numbers 1–4 indicate the sequence regions in centromeres. All data were calculated by a 500 kb sliding window and 100 kb step size.

Through ONT ultralong‐read sequencing, eight of the ten chromosomes were assembled as gap‐free, and the remaining two centromeres were significantly improved in Chiifu v4.0 (Table S1). It provides an unprecedented opportunity to study the landscape of centromeres in *B. rapa*. To identify the location and sequence of centromeres in our new assembly, we used the enrichment of Cent‐SRs, including *CentBr1*, *CentBr2, CRB* and *TR805* (Koo *et al*., 2011; Lim *et al*., 2005, 2007), which was directly associated with BrCENH3 proteins in *B. rapa* (Wang *et al*., 2011a). Of the ten centromeres, two centromeres on chromosomes A03 and A05 were enriched for *CenBr2*, while the other eight centromeres were enriched for *CenBr1* (Figure S6). Although the chromosome name of previous fluorescence in situ hybridization (FISH) experiments did not correspond well with that of Chiifu v4.0, eight centromeres enriching for *CenBr1* and two for *CenBr2* were consistent with the previous FISH experiments (Lim *et al*., 2007). Sequence analysis revealed that 94.23% (65.01 Mb/68.99 Mb) of the centromeric region was occupied by LTRs (Figure 3a; Table S2). Among these 555 centromeric genes of Chiifu v4.0, 17.66% (98/555) of them were transcribed, much lower than the gene transcription ratio of the whole genome (45.57%, 21 659/47 531).

To better understand the long‐range organization of centromeres, we generated a heatmap showing the pairwise sequence identity along the centromeres. The results showed that centromeres were disrupted into different regions in the centromeric sequences in Chiifu v4.0 (Figures 3b,c and S7). In *A. thaliana*, the centromeres CEN4 and CEN5 are invaded by *ATHILA* LTRs (Naish *et al*., 2021). In Chiifu v4.0, we identified 3256 full‐length long terminal repeat retrotransposons (FL‐LTR‐RTs) for the whole genome and grouped them into 12 families based on repeat domain protein homology (Figure S8; Table S3). We detected 974 FL‐LTR‐RTs in centromeres (Table S3), and 34.49% (336/974) contained Cent‐SRs. Notably, among the 12 FL‐LTR‐RTs families, 539 *ALE* (*Copia*) and 281 *CRM* (*Gypsy*) LTRs were specifically increased in copy number within these invaded regions in the centromeres (Figures 3b,c and S7). These results indicated that the centromeres were mainly invaded by *ALE* and *CRM* LTRs, further shaping the centromere structures in *B. rapa*.

### Diversity of centromeres among *B. rapa* genomes

In our newly assembly Chiifu v4.0, eight complete centromeres with no gap were assembled, namely CentA01 (7.06 Mb), CentA02 (7.10 Mb), CentA03 (4.44 Mb), CentA04 (7.09 Mb), CentA06 (4.00 Mb), CentA07 (10.75 Mb), CentA09 (5.10 Mb) and CentA10 (11.75 Mb). For comparison with other *B. rapa* genome assemblies, the same criteria of Chiifu v4.0 were used to define the centromere boundaries of other genome assemblies (Table S1). The results showed that all centromeres have gaps in other *B. rapa* genome assemblies, except for the centromere of chromosome A08 in NHCC001 (Figures 2 and S5; Table S1). Furthermore, we found that Chiifu v4.0, assembly “A03”, PC‐fu, ECD04 and Z1 v2 had significantly more assembled Cent‐SRs than NHCC001 and ZYCX. However, PC‐fu had many Cent‐SRs on the end of chromosomes, indicating the misassembled Cent‐SRs in its genome (Figure S6; Table S4). Thus, Chiifu v4.0, assembly “A03”, ECD04 and Z1 v2 were used for our subsequent analysis.

We found that the centromere length varied significantly among different *B. rapa* genomes. To avoid making an incorrect conclusion due to the incomplete assembly, we only compared the eight gap‐free centromeres of Chiifu v4.0 with the orthologous centromeres of the other three assemblies (assembly “A03”, ECD04 and Z1 v2). After removing gaps, CentA04 of assembly “A03” (8.76 Mb) and CentA06 of Z1 v2 (5.56 Mb) were still longer than the orthologous centromeres of Chiifu v4.0 (7.09 Mb of CentA04; 4.00 Mb of CentA06; Table S1).

**Figure 4:**
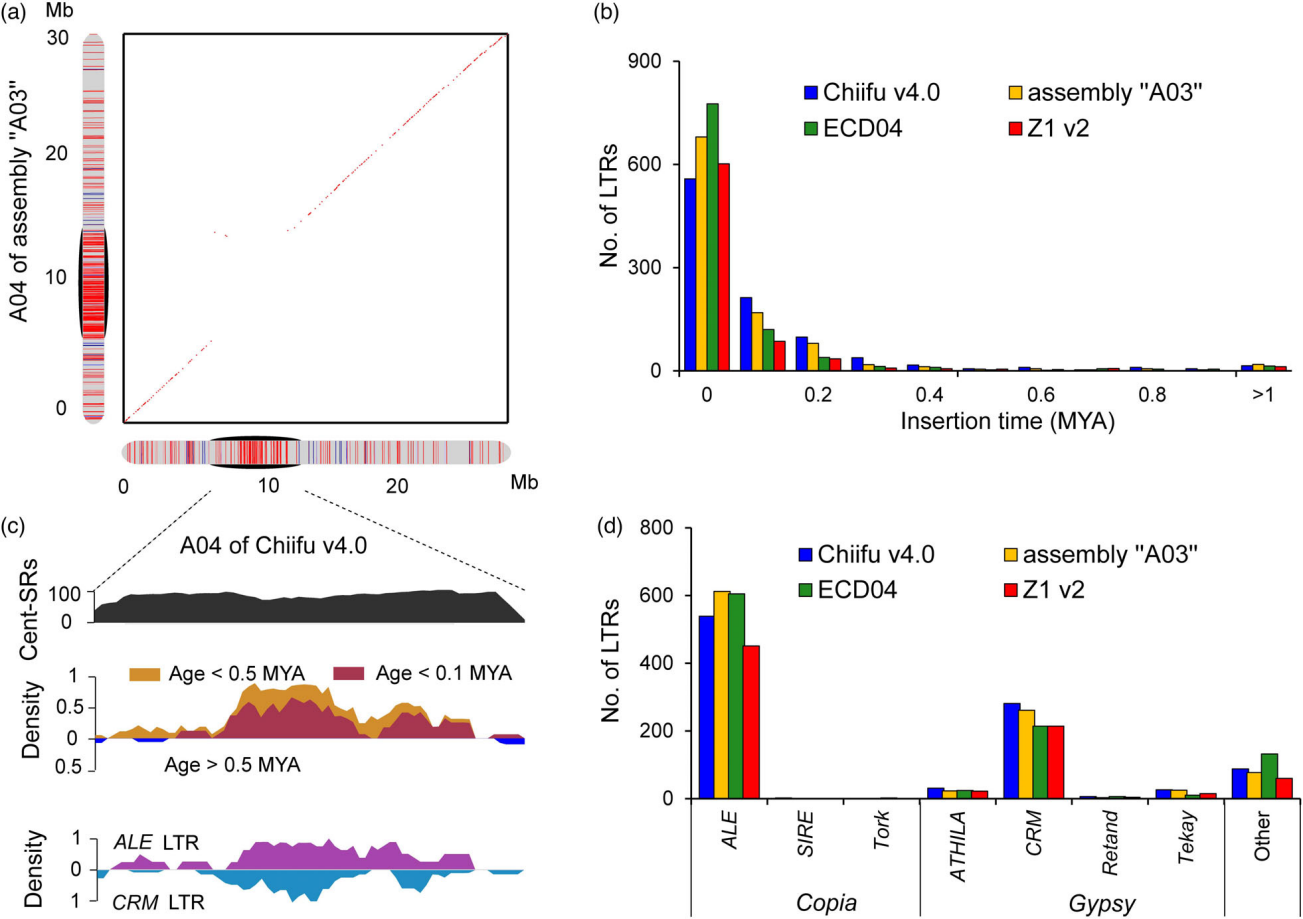
Comparison of centromeres among *Brassica rapa* genome assemblies. (a) Collinearity of chromosome A04 of Chiifu v4.0 and assembly “A03”. Black ovals indicate centromeres. (b) Insertion time of FL‐LTR‐RTs in centromeres among different assemblies. (c) Distribution of features on the centromere CentA04 in Chiifu v4.0, including Cent‐SRs, LTR age and family. The density was calculated using a 500 kb sliding window and 100 kb step size. (d) Copy number of FL‐LTR‐RT families in centromere regions of *B. rapa* genome assemblies.

Chromosomal collinearity analysis showed that the orthologous centromeres had little or no sequence collinearity among the *B. rapa* assemblies. For example, unlike the chromosomal arms, the centromeres CentA04 between Chiifu v4.0 and assembly “A03” had almost no sequence collinearity (Figure 4a). Little sequence collinearity was also observed when we compared the other seven complete centromeres of Chiifu v4.0 with the orthologous centromeres of assembly “A03”, ECD04 and Z1 v2 (Figure S9). Together, these results indicated that the centromeres are highly variable among different *B. rapa* genomes.

### Rapidly amplified LTRs drove the evolution of centromeres

Sequence analysis revealed that TEs contributed 92.90%–97.17% of centromere sequences in different *B. rapa* genomes (Table S2). We further annotated the FL‐LTR‐RTs in assembly “A03”, ECD04 and Z1 v2. A total of 1001, 993 and 767 FL‐LTR‐RTs were identified in the centromeric regions of assembly “A03”, ECD04 and Z1 v2, which was similar to the quantity of FL‐LTR‐RTs in the centromeres of Chiifu v4.0 (974) (Figure 4b; Table S3). Analysing the insertion time of FL‐LTR‐RTs in centromeres showed that 78.83%–86.04% of FL‐LTR‐RTs were amplified ≤0.5 MYA and 38.57%–57.78% were amplified ≤0.1 MYA in Chiifu v4.0, assembly “A03”, ECD04 and Z1 v2. In comparison, 5.64%–7.86% were amplified >1 MYA in the centromeres of Chiifu v4.0, assembly “A03”, ECD04 and Z1 v2 (Figure 4b). Furthermore, we detected 539, 612, 605 and 451 *ALE* LTRs and 281, 261, 214 and 214 *CRM* LTRs in the centromeres of Chiifu v4.0, assembly “A03”, ECD04 and Z1 v2, respectively (Figure 4d). These findings suggested that LTRs are shared but exhibit different ages and copy numbers in the centromeres of *B. rapa*.

**Figure 5:**
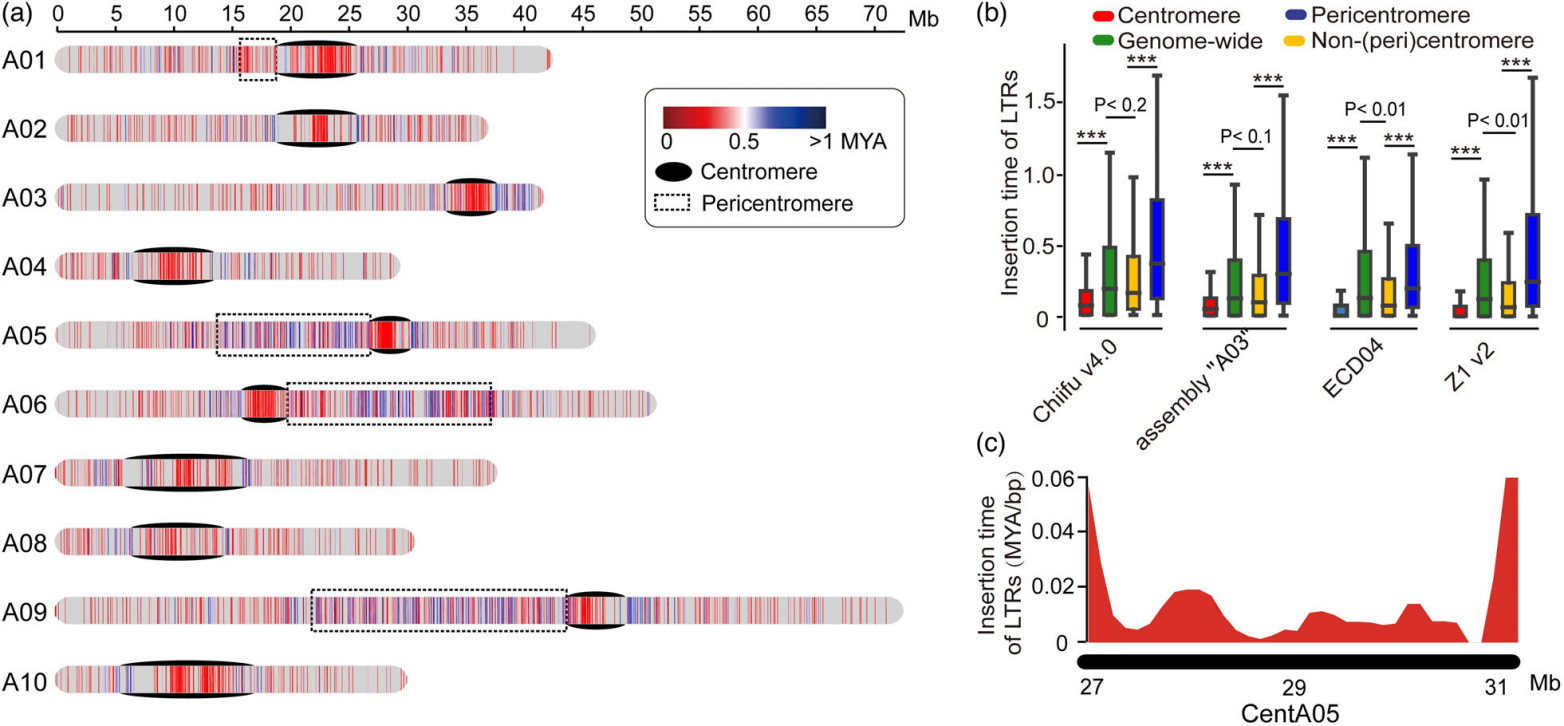
Young LTRs in the centromeres of *Brassica rapa*. (a) Age distribution of FL‐LTR‐RTs in Chiifu v4.0. Black ovals indicate centromeres, and dashed rectangles indicate pericentromeres. (b) Comparison of the insertion time of FL‐LTR‐RTs for the centromeres, pericentromeres, non‐(peri)centromeres and whole genome in different *B. rapa* genome assemblies. The *P* values were calculated using a two‐sided *t‐*test. ‘***’ indicates *P* < 0.001. (c) The density of insertion time of FL‐LTR‐RTs in centromere CentA05 of Chiifu v4.0. The density of insertion time was calculated using a 500 kb sliding window and 100 kb step size.

According to a recent study (Perumal *et al*., 2020), we defined FL‐LTR‐RTs with age ≤0.5 MYA as young LTRs and age >0.5 MYA as old LTRs. The age distribution analysis of FL‐LTR‐RTs showed that the centromere regions were enriched for young LTRs in *B. rapa* (Figures 5a and S10). Further comparison of the insertion time of LTRs in different chromosomal regions in Chiifu v4.0 showed that FL‐LTR‐RTs in centromeres were significantly younger (0.14 MYA on average) than those of the whole genome (0.32 MYA on average; Figure 5b). We found that LTRs in the center part of centromeres were much younger than other portions of centromeres in Chiifu v4.0 (Figures 5c and S11). Furthermore, we identified 83 nested insertion events of FL‐LTR‐RTs in Chiifu v4.0 (Table S5), which were much fewer than that of *B. nigra* (262 events, Perumal *et al*., 2020). Together, our results suggested that the LTRs were rapidly amplified in centromeres, which could drive the evolution of centromeres in *B. rapa*.

### Old LTRs were enriched in *B. rapa* pericentromeres

Due to being highly repetitive, pericentromeres have rarely been studied in *Brassica* genomes. To determine the pericentromeres in *B. rapa*, the Peri‐SRs, including *PCRBr* and *TR238* (Lim *et al*., 2007), were used to delimit the boundaries and sizes of pericentromeres in Chiifu v4.0, assembly “A03”, ECD04 and Z1 v2. Peri‐SRs were detected on chromosomes A01, A05, A06 and A09 in *B. rapa* genomes (Figures S6 and S12; Table S6). Our findings of four chromosomes with Peri‐SRs were consistent with the results of previous FISH experiments (Lim *et al*., 2007). Sequence analysis indicated that 90.27% (12 608/13 966) of rRNA sequences were located in the pericentromeric regions in Chiifu v4.0 (Figure S13). Among these 773 FL‐LTR‐RTs in pericentromeres, 37.00% (286) were *CRM*, and 34.41% (266) were *Tekay* LTRs, indicating that pericentromeres were invaded by *CRM* and *Tekay* LTRs (Figure S14). For these 263 pericentromeric genes, 19.39% (51/263) were found to be transcribed, which was much lower than that of the whole genome (45.57%) but slightly higher than that of centromeres (17.66%).

**Figure 6:**
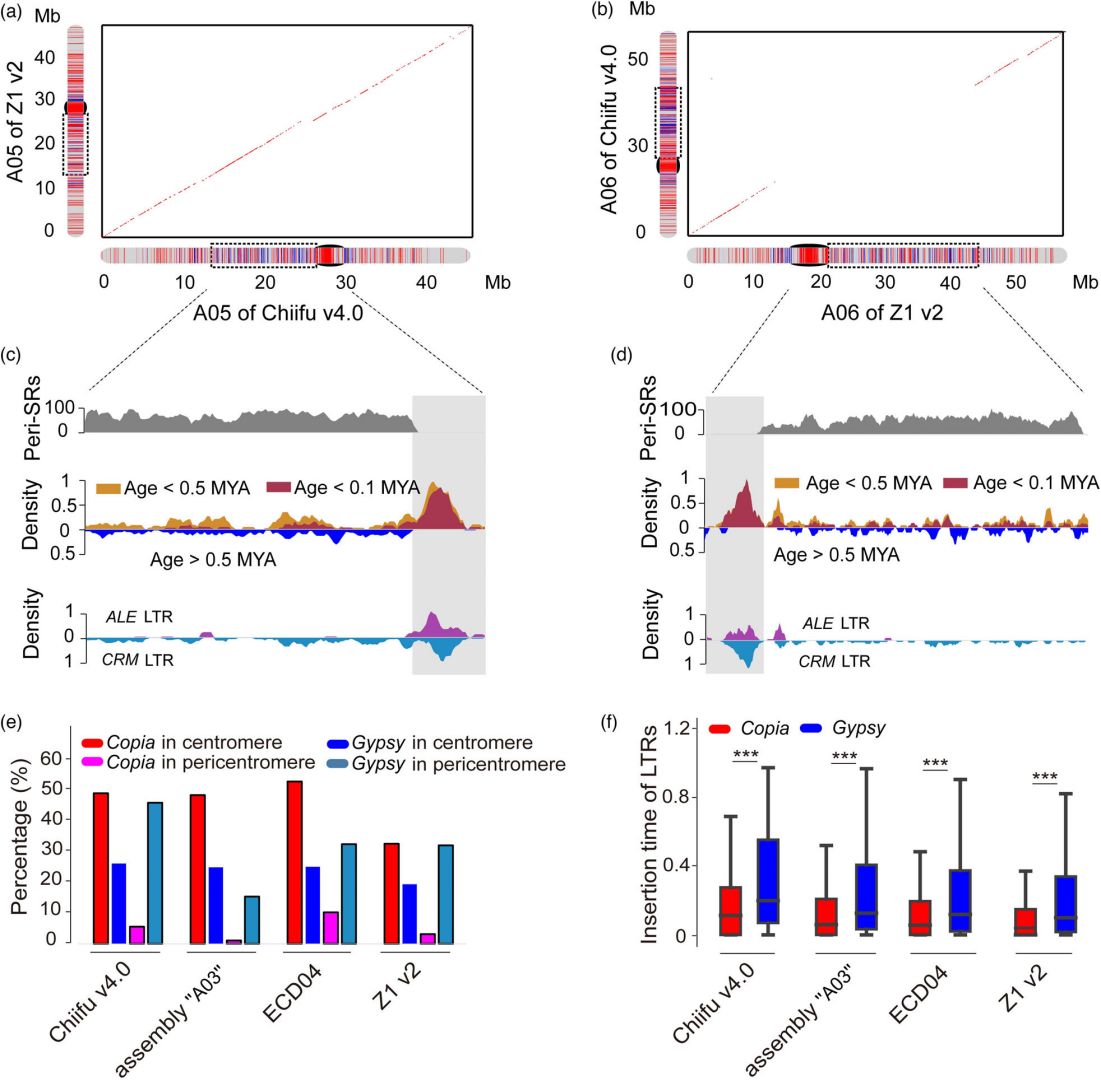
Old LTRs in the pericentromeres of *Brassica rapa*. (a) Collinearity of chromosome A05 of Chiifu v4.0 and Z1 v2. (b) Collinearity of chromosome A06 of Z1 v2 and Chiifu v4.0. Black rectangles indicate the centromeres, and dashed rectangles indicate the pericentromeres. (c, d) Distribution of various features in the pericentromere regions of chromosome A05 of Chiifu v4.0 (c) and A06 of Z1 v2 (d), including Peri‐SRs, LTR age and family. Grey blocks indicate the centromere regions. The density was calculated using a 500 kb sliding window and 100 kb step size. (e) Percentage of *Copia* and *Gypsy* LTRs in centromeres and pericentromeres. (f) Insertion time of the *Copia* and *Gypsy* LTRs. The *P* values were calculated using a two‐sided *t‐*test. ‘***’ indicates *P* < 0.001.

After comparing the insertion time of LTRs between pericentromeres and other chromosomal regions in Chiifu v4.0, we found that the insertion time of FL‐LTR‐RTs in pericentromeres was significantly older (0.51 MYA on average) than those of the whole genome (0.32 MYA on average) and further much older than those in centromeres (0.14 MYA on average) (Figure 5b). Similar patterns were found in assembly “A03”, ECD04 and Z1 v2 (Figure 5b). Comparing the LTRs between pericentromeres and centromeres revealed that pericentromeres were enriched for *Gypsy* LTRs while centromeres were enriched for more *Copia* than *Gypsy* LTRs (Figures 6c–e and S15). Furthermore, the insertion time of *Gypsy* LTRs (0.29–0.38 MYA on average) was prominent older than that of *Copia* LTRs (0.14–0.22 MYA on average; Figure 6f), which was probably why the LTRs in pericentromeres were older than those in the centromeres of *B. rapa*.

We identified a novel pericentromeric repeat based on the FL‐LTR‐RT sequences in *B. rapa*. We used self‐self‐blast of the 773 pericentromere‐enriched FL‐LTR‐RTs and filtered out the repeats that occurred fewer than 50 times. Then we blasted the remaining sequences to the pericentromere sequences and kept the repeats which hit on the pericentromeres over 1000 times. After filtering, we identified a novel repeat, termed *PCR630*, which is 630 bp in length with 1550 hits on pericentromeres. There was no sequence similarity when it was compared with either *PCRBr* (1022 hits on pericentromeres) or *TR238* (87 407 hits on pericentromeres). We further found that *PCR630* had a similar pericentromere‐specific distribution pattern as *PCRBr and TR238* in *B. rapa* (Figure S16).

## Discussion

The *B. rapa* Chiifu genome has been the most widely used reference genome in the *Brassica* research community since it was published in 2011 (citations >2000 times based on Google Scholar of November 2022; Wang *et al*., 2011b; Cai *et al*., 2017; Zhang *et al*., 2018). However, Chiifu 3.0, the current reference genome of *B. rapa*, still has 407 gaps and a relatively short contig N50 of 1.45 Mb (Zhang *et al*., 2018). In this study, we presented a near‐complete genome assembly, *B. rapa* Chiifu v4.0, using ONT sequencing and Hi‐C technologies. The contig N50 of Chiifu v4.0 (38.26 Mb) is the largest among the published *Brassica* genomes up to November 2022. Long‐read sequencing provides more comprehensive genome coverage. In Chiifu v4.0, eight telomere‐to‐telomere chromosomes were well assembled, and only two gaps were located on the centromeres of the other two chromosomes (Table 1). We identified 18 telomeres on nine chromosomes by screening the telomere‐specific repeat (*BrSTR*, Koo *et al*., 2011; Figure S17). Compared with Chiifu v3.0, the new assembly added ~71.45 Mb novel sequences, ~10% more repeats, and 298 novel genes, almost all located in the centromeres and pericentromeres. The near‐complete reference genome, *B. rapa* Chiifu v4.0, representing the highest completeness, reliability and quality, will drive the future discovery of genome structure and functional genes in *Brassica*.

Centromeres show rapid divergence among *B. rapa* genomes. Centromeres comprise highly repetitive elements, which are structures essential for maintaining chromosome integrity during cell division and ensuring the fidelity of their inheritance of chromosome complements (Comai *et al*., 2017). Although functions of centromeres are highly conserved in chromosome segregation among eukaryotes, centromeres evolve with high variability and show little or no collinearity in sequence and DNA composition even among closely related species (Gao *et al*., 2015; Talbert and Henikoff, 2022). In this study, the size and sequence of centromeres on orthologous chromosomes varied among different *B. rapa* genomes (Figures 4a and S6), indicating that centromeres are highly variable not only among very closely related species but also between distinct accessions within the same species. These results are likely related to the low recombination ratio in centromeric regions (Zhang *et al*., 2018), which may prevent the exchange of centromeric sequences and result in high variability in centromeres.

Rapidly amplified LTRs drive the evolution of centromeres. In *B. rapa*, centromeres were mainly invaded by *ALE* and *CRM* LTRs in *B. rapa* (Figures 3 and S7), suggesting that these two LTR families might play an essential role in the rapid evolution of centromeres (Figure 4d). It was noted that *Arabidopsis* centromeres had been invaded by *ATHILA* LTRs (Naish *et al*., 2021), and *ALE* LTRs had significantly increased in the centromeres of *B. nigra* (Perumal *et al*., 2020). In this study, we found that LTRs were shared in the centromeres in different *B. rapa* genomes, but the abundances and ages of LTRs were vastly divergent, suggesting that the rapidly changed LTRs could drive the evolution of centromeres in *B. rapa*. In the future, more studies are required to fully establish the role of the recently amplified LTRs in centromeres in *B. rapa*.

The old LTRs were enriched in the pericentromeres. Previous studies have shown that LTRs in centromeres are younger than those of other chromosomal regions in *B. nigra* (Perumal *et al*., 2020) and cotton (Yang *et al*., 2021). The present study also showed younger LTRs in centromeres of *B. rapa* (Figure 5a). Interestingly, LTRs in pericentromeres were much older (0.37 MYA on average) than those in centromeres in *B. rapa* (Figure 5b). In humans, the mutation rate of centromeric sequences is accelerated more than 2.2‐fold compared with other portions of the chromosome (Altemose *et al*., 2022; Logsdon *et al*., 2021). It could imply that the rapid amplification of young LTRs in the centromeres could force the old LTRs out, leading to the residence of relatively old LTRs in the flanking pericentromeres in *B. rapa*.

Together, our near‐complete genome assembly, *B. rapa* Chiifu v4.0, provides a critical genome resource for the *Brassica* research community and reveals the rapid evolution of centromeres in *B. rapa*. Such resources will provide a solid foundation for elucidating the genome structure and functions of *Brassica* species.

## Materials and methods

### Genome sequencing and *de novo* assembly

*Brassica rapa* L. ssp. *pekinensis* inbred line (Chiifu‐401‐42) was used for whole‐genome sequencing in this study. In total, 500 mg of frozen leaf tissues were used to generate high‐quality genomic DNA. For the Chiifu genome, the R9.4.1 (SQK‐LSK110) genomic library was prepared following the nanopore protocol (https://community.nanoporetech.com/protocols). The libraries were then sequenced on a Promethion platform, and MinKnow with Guppy (v5.0.16) was used for base calling with default parameters. A total of 90.24 Gb of ONT long reads with ~180 × coverage was generated, including ~64× coverage of ultralong reads (>50 kb). The ONT read N50 was 40 kb, and the most extended read was 487 325 bp.

Subsequently, the raw ONT data were filtered for quality at Q10, and the resulting reads were de novo assembled using NextDenovo (v2.5, https://github.com/Nextomics/NextDenovo) with parameters: “read_cutoff = 5k” and “seed_cutoff = 75 000”. The raw ONT reads were error‐corrected using Canu (v1.5; Koren *et al*., 2017) with default parameters. The resulting contigs were polished using three iterations of Racon (v1.4.3; Vaser *et al*., 2017) with correct ONT reads and two iterations of Pilon (v1.22; Walker *et al*., 2014) with Illumina reads obtained from BRAD (http://brassicadb.org; Chen *et al*., 2022).

### Contigs scaffolding and gap filling

The *B. rapa* Hi‐C data from our previous study were used to correct and scaffold polished contigs (Zhang *et al*., 2018). We first aligned these Hi‐C reads to raw contigs using bowtie2 (v2.3.3; Langmead and Salzberg, 2012). Contact maps for all contigs produced by HiC‐Pro (v3.1.0; Servant *et al*., 2015) were drawn using the ggplot2 package (http://ggplot2.org/). We then checked the interaction signals for each contig with the others and split them when they had a strong signal with distant sequences. Finally, the corrected contigs were used as input for scaffolding by 3D‐DNA (v180922; Dudchenko *et al*., 2017) with default parameters. Scaffolds were manually checked and refined with Juicebox (v1.11.08; Durand *et al*., 2016). The gaps in these scaffolds were closed by TGS_GapCloser (v1.1.1; Xu *et al*., 2020) with corrected ONT long reads. We finally filled the gaps with the corrected ONT, BAC (PRJEA28961) and corrected PacBio reads (Zhang *et al*., 2018) of *B. rapa* Chiifu‐401‐42 using a python script (https://github.com/zhangleiworld/gapfill_by_reads).

### Genome annotation

Genes were lifted from Chiifu v3.5 with Liftoff (v1.5.1; Shumate and Salzberg, 2021). MAKER‐P (v3; Campbell *et al*., 2014) was used to annotate the newly assembled regions in Chiifu v4.0. TEs were identified using EDTA (v1.9.6; Ou *et al*., 2019). FL‐LTR‐RTs were identified using LTR_retriever (v2.9.0; Ou and Jiang, 2018b) as described previously (Perumal *et al*., 2020) and further classified by TEsorter (v1.1.1; Zhang *et al*., 2022a). The insertion time of the FL‐LTR‐RTs was calculated as previously described (Liu *et al*., 2014). The FL‐LTR‐RTs were manually analysed to identify nested TE insertion following a previous study (Perumal *et al*., 2020). The LTR assembly index score was calculated by LAI (vbeta3.2; Ou *et al*., 2018a).

### Identification of synteny between Chiifu v4.0 and other assemblies

Chiifu v4.0 was aligned to other *B. rapa* assemblies using Mummer (v4.0.0beta2; Marçais *et al*., 2018) with parameter settings “‐‐mum ‐c 5 000 ‐l 2 000”. Then, we used the “delta‐filter‐1” parameter with the one‐to‐one alignment block option to filter the alignment results. Further, “show‐coords” were used to show the synteny's coordinate between Chiifu v4.0 and other *B. rapa* assemblies.

### Identification of centromeres, pericentromeres, telomeres and rRNA

We used LASTZ (v1.04.00; http://www.bx.psu.edu/~rsharris/lastz/) to align the Cent‐SRs (*CentBr1*, *CentBr2*, *CRB* and *TR805*) and Peri‐SRs (*PCRBr* and *TR238*) to the reference genome (Koo *et al*., 2011; Lim *et al*., 2005, 2007). The signals of the Cent‐SRs and Peri‐SRs were used as evidence supporting the localization of the centromeres and pericentromeres in Chiifu v4.0. To identify the telomeres in *B. rapa*, the telomere‐specific sequence *BrSTR* (Koo *et al*., 2011) was aligned with Chiifu v4.0. The rRNA sequences were predicted by Infernal (v1.1.2; Nawrocki and Eddy, 2013) using the Rfam database. The same methods were used to identify the centromeres, pericentromeres, telomeres and rRNA sequences in the other *B. rapa* genome assemblies.

### Identification of the novel pericentromeric repeat

The novel pericentromeric repeat was identified using a self‐self‐blast of the FL‐LTR‐RT sequences in pericentromeres of Chiifu v4.0. BLASTN was run using an e‐value cutoff of 1e‐5. Any repeat that occurred over 50 times and hit on the pericentromeres more than 1000 times was defined as the novel pericentromeric repeat. The sequence of novel pericentromeric repeat was listed in Table S7.

## Conflicts of interest

No conflict of interest was declared.

## Author contributions

X.W. designed the project; L.Z., J.W. and J.L. prepared materials and performed the experiments; L.Z., Z.Z., H.C. and X.W. performed the data analysis; L.Z. and X.W. wrote the manuscript; J.W. and J.L. revised the manuscript. All authors read and approved the final manuscript.

## Supporting information


**Figure S1** Whole‐genome Hi‐C heatmap of *Brassica rapa* Chiifu v4.0 at 100 kb resolution.
**Figure S2** Comparison of the heatmap and gaps of the conflict regions between *Brassica rapa* Chiifu v4.0 and v3.0.
**Figure S3** The position of the additional sequences in *Brassica rapa* Chiifu v4.0 relative to Chiifu v3.0.
**Figure S4** The gap positions of *Brassica rapa* Chiifu v4.0.
**Figure S5** Chromosome collinearity between *Brassica rapa* Chiifu v4.0 and the other assemblies.
**Figure S6** The distribution of Cent‐SRs and Peri‐SRs in *Brassica rapa* genome assemblies.
**Figure S7** Heatmap shows pairwise sequence identity between all non‐overlapping 10 kb regions of centromeres in *Brassica rapa* Chiifu v4.0.
**Figure S8** Annotation of FL‐LTR‐RTs in different *Brassica rapa* genome assemblies.
**Figure S9** Centromere collinearity between *Brassica rapa* Chiifu v4.0 and other assemblies.
**Figure S10** The age distribution of FL‐LTR‐RTs in *Brassica rapa* genome assemblies.
**Figure S11** The density of insertion time of FL‐LTR‐RTs in centromeres of *Brassica rapa* Chiifu v4.0.
**Figure S12** Characterization of the pericentromeres in *Brassica rapa* Chiifu v4.0.
**Figure S13** The distribution of rRNA sequences in *Brassica rapa* genome assemblies.
**Figure S14** Heatmap shows pairwise sequence identity between all non‐overlapping 10 kb regions of pericentromeres in *Brassica rapa* Chiifu v4.0.
**Figure S15** The family distribution of FL‐LTR‐RTs in *Brassica rapa* genome assemblies.
**Figure S16** The distribution of *PCR630* in *Brassica rapa* genome assemblies.
**Figure S17** The distribution of telomere‐specific repeat in *Brassica rapa* genome assemblies.

**Table S1** The location of centromeres in *Brassica rapa* genome assemblies.
**Table S2** Summary of TEs and LTRs in the centromeres of *Brassica rapa* genome assemblies.
**Table S3** Summary of FL‐LTR‐RTs in *Brassica rapa* genome assemblies.
**Table S4** Summary of Cent‐SRs and Peri‐SRs in *Brassica rapa* genome assemblies.
**Table S5** Summary of nested LTRs events in *Brassica rapa* genome assemblies.
**Table S6** The location of pericentromeres in *Brassica rapa* genome assemblies.
**Table S7** The sequence of *PCR630* in *Brassica rapa*.

## Data Availability

The raw ONT reads are freely available through the Genome Sequence Archive under accession number CRA008441 (https://ngdc.cncb.ac.cn/gsa/). The genome sequences are freely available through the BRAD website (http://39.100.233.196:82/download_genome/Brassica_Genome_data/Brara_Chiifu_V4.0/). All other data generated or analysed during this study are included in this published article and its supplementary information files.
