# Supplementary material for: A near‐complete genome assembly of Brassica rapa provides new insights into the evolution of centromeres
Source: Plant Biotechnol J. 2023 Feb 2;21(5):1022–32. doi: 10.1111/pbi.14015 (PMC10106856; doi:10.1111/pbi.14015)
Supplement: Supplementary file 1 — Figure S1 Whole‐genome Hi‐C heatmap of Brassica rapa Chiifu v4.0 at 100 kb resolution. Figure S2 Comparison of the heatmap and gaps of the conflict regions between Brassica rapa Chiifu v4.0 and v3.0. Figure S3 The position of the additional sequences in Brassica rapa Chiifu v4.0 relative to Chiifu v3.0. Figure S4 The gap positions of Brassica rapa Chiifu v4.0. Figure S5 Chromosome collinearity between Brassica rapa Chiifu v4.0 and the other assemblies. Figure S6 The distribution of Cent‐SRs and Peri‐SRs in Brassica rapa genome assemblies. Figure S7 Heatmap shows pairwise sequence identity between all non‐overlapping 10 kb regions of centromeres in Brassica rapa Chiifu v4.0. Figure S8 Annotation of FL‐LTR‐RTs in different Brassica rapa genome assemblies. Figure S9 Centromere collinearity between Brassica rapa Chiifu v4.0 and other assemblies. Figure S10 The age distribution of FL‐LTR‐RTs in Brassica rapa genome assemblies. Figure S11 The density of insertion time of FL‐LTR‐RTs in centromeres of Brassica rapa Chiifu v4.0. Figure S12 Characterization of the pericentromeres in Brassica rapa Chiifu v4.0. Figure S13 The distribution of rRNA sequences in Brassica rapa genome assemblies. Figure S14 Heatmap shows pairwise sequence identity between all non‐overlapping 10 kb regions of pericentromeres in Brassica rapa Chiifu v4.0. Figure S15 The family distribution of FL‐LTR‐RTs in Brassica rapa genome assemblies. Figure S16 The distribution of PCR630 in Brassica rapa genome assemblies. Figure S17 The distribution of telomere‐specific repeat in Brassica rapa genome assemblies. [file PBI-21-1022-s002.docx]

**Supplementary figures**


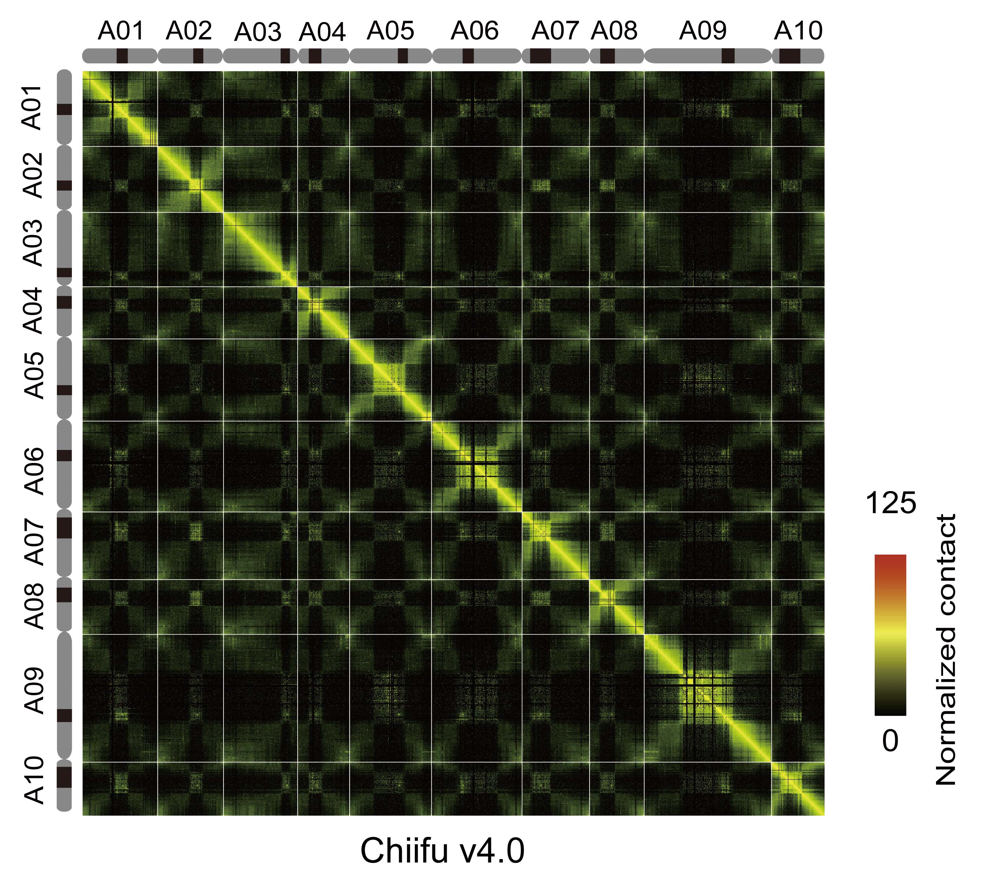


**Figure S1** **Whole-genome Hi-C heatmap of *Brassica rapa* Chiifu v4.0 at 100 kb resolution.**


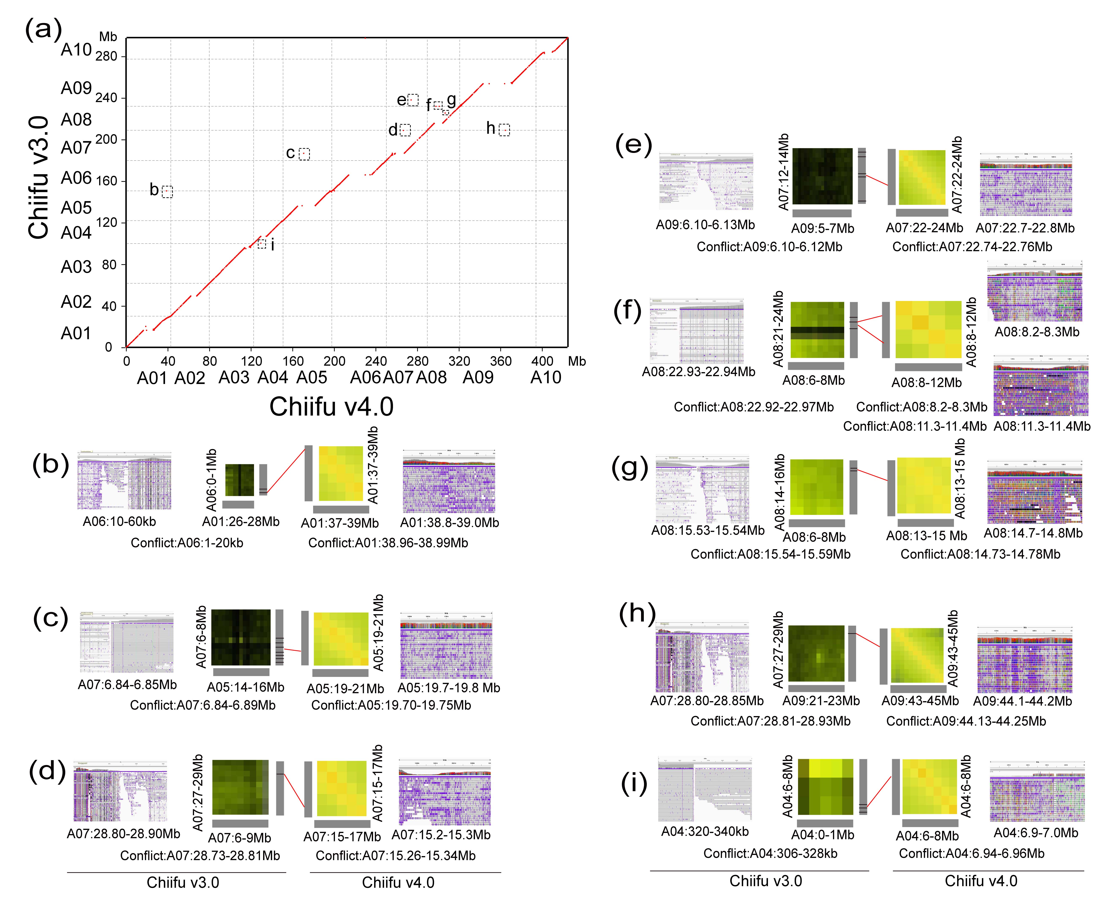


**Figure S2** **Comparison of the heatmap and gaps of the conflict regions between *Brassica rapa* Chiifu v4.0 and v3.0.** IGV shows the ONT reads coverage of the conflict regions of Chiifu v3.0 and v4.0. Hi-C heatmap shows the conflict regions of Chiifu v4.0 and v3.0. Hi-C heatmap was constructed at 100 kb resolution. Black lines indicate the gaps on chromosomes of Chiifu v3.0. Red lines indicate the syntenic regions between Chiifu v4.0 and v3.0.


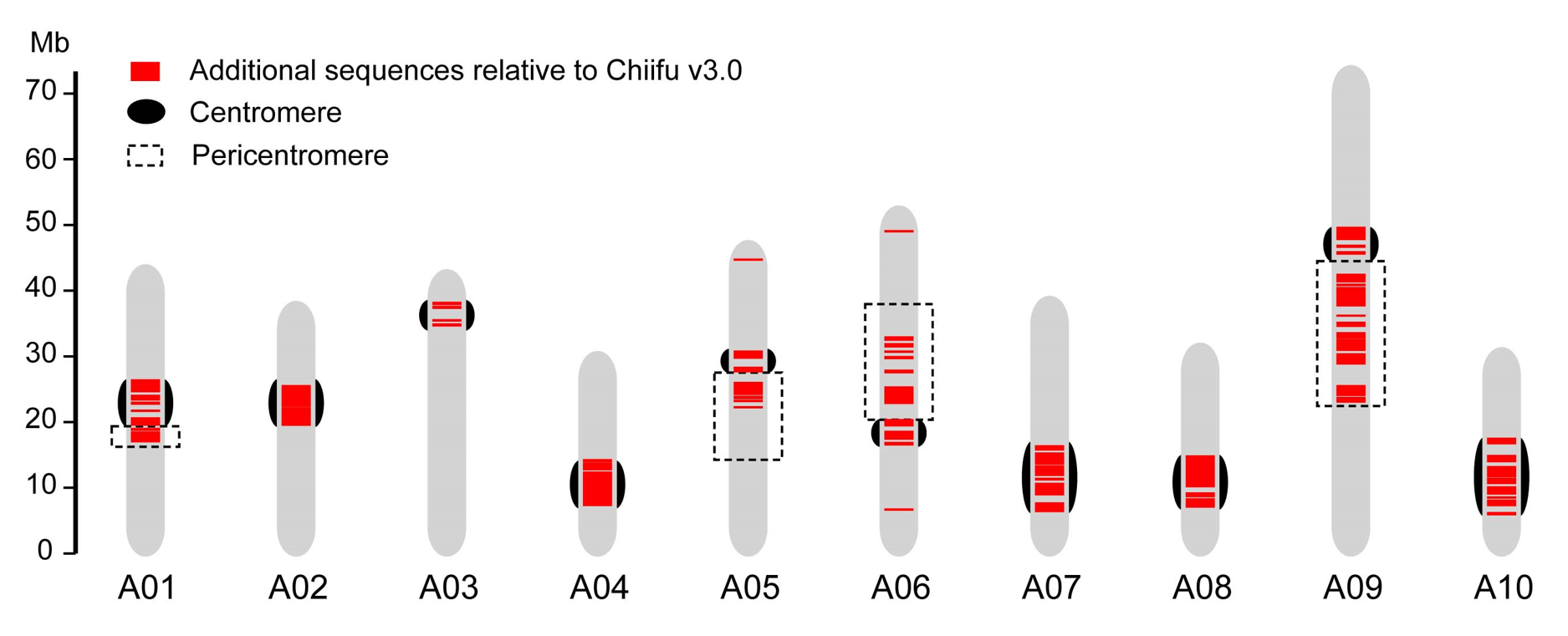


**Figure S3** **The position of the additional sequences in *Brassica rapa* Chiifu v4.0 relative to Chiifu v3.0.** Red rectangles indicate the additional sequences relative to Chiifu v3.0. Black ovals indicate the centromeres, and dashed rectangles indicate the pericentromeres.


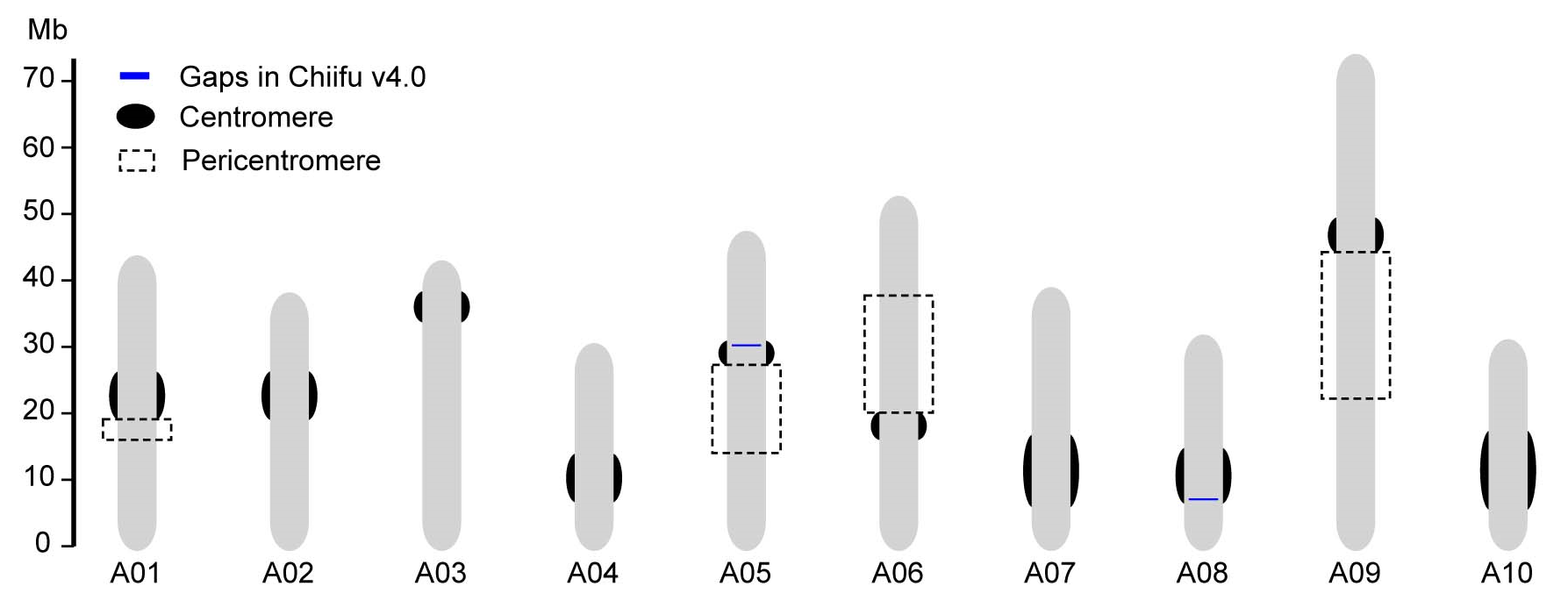


**Figure S4 The gap positions of *Brassica rapa* Chiifu v4.0**. Blue lines indicate the gaps in the chromosomes. Black ovals indicate the centromeres, and dashed rectangles indicate the pericentromeres.


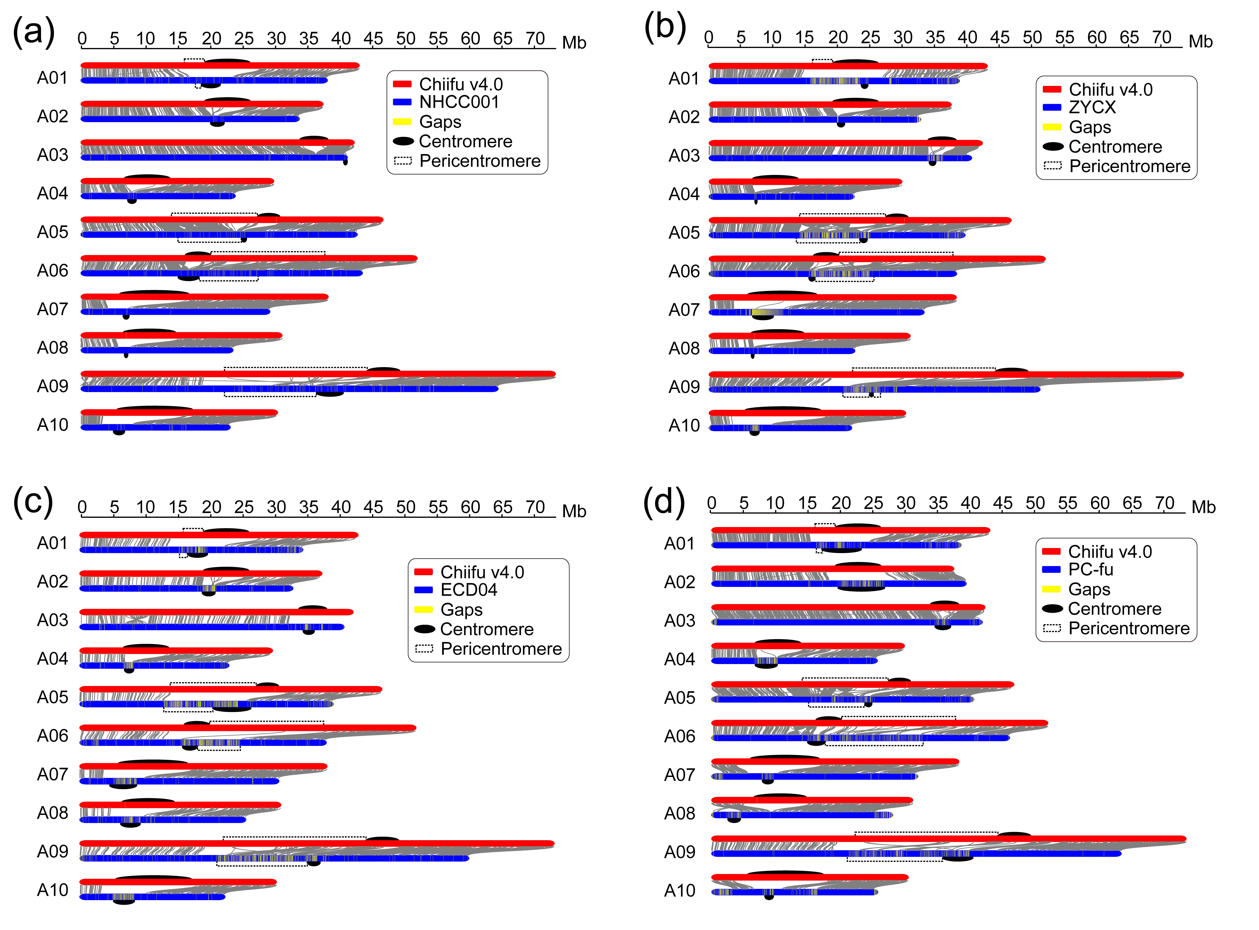


**Figure S5** **Chromosome collinearity between *Brassica rapa* Chiifu v4.0 and the other assemblies.** Grey lines link the collinear regions, and gaps are shown in yellow blocks. Black blocks indicate centromeres, and dashed blocks indicate pericentromeres.


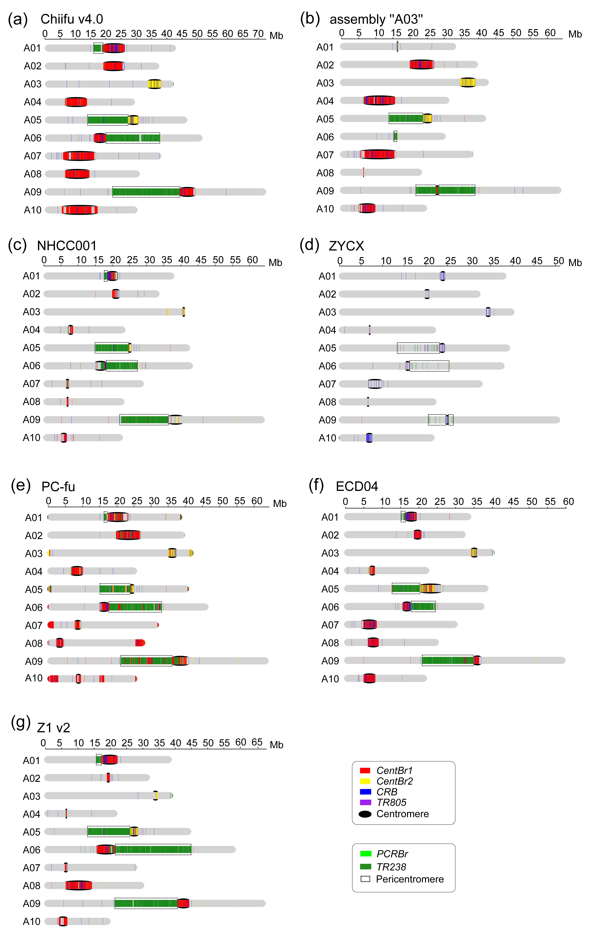


**Figure S6** **The distribution of Cent-SRs and Peri-SRs in *Brassica rapa* genome assemblies.** Black blocks indicate centromeres, and dashed blocks indicate pericentromeres.


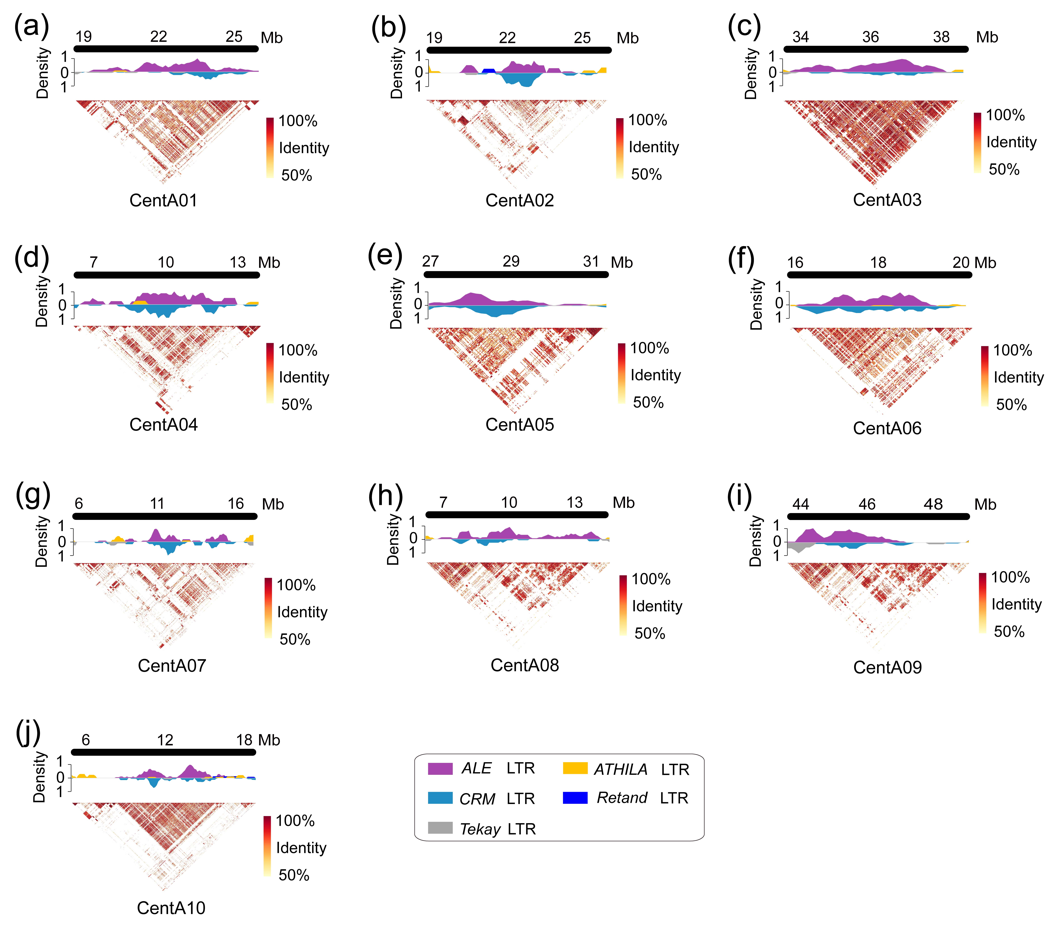


**Figure S7 Heatmap shows pairwise sequence identity between all non-overlapping 10 kb regions of centromeres in *Brassica* *rapa* Chiifu v4.0.**


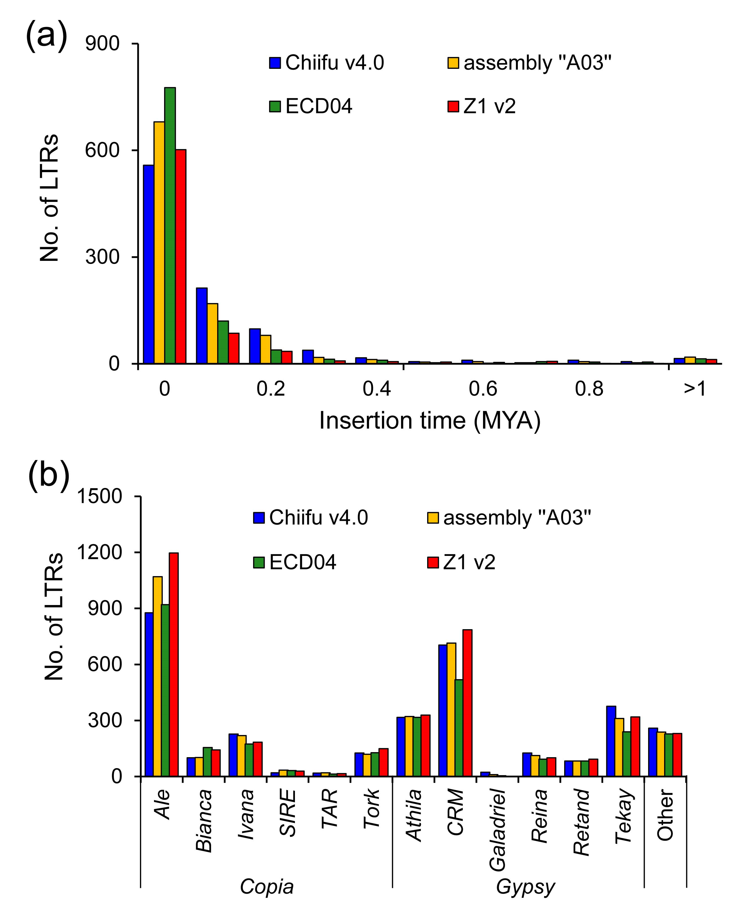


**Figure S8** **Annotation of FL-LTR-RTs in different *Brassica rapa* genome assemblies.** **(a)** Age distribution of FL-LTR-RTs. **(b)** Copy number of FL-LTR-RTs from 11 families.


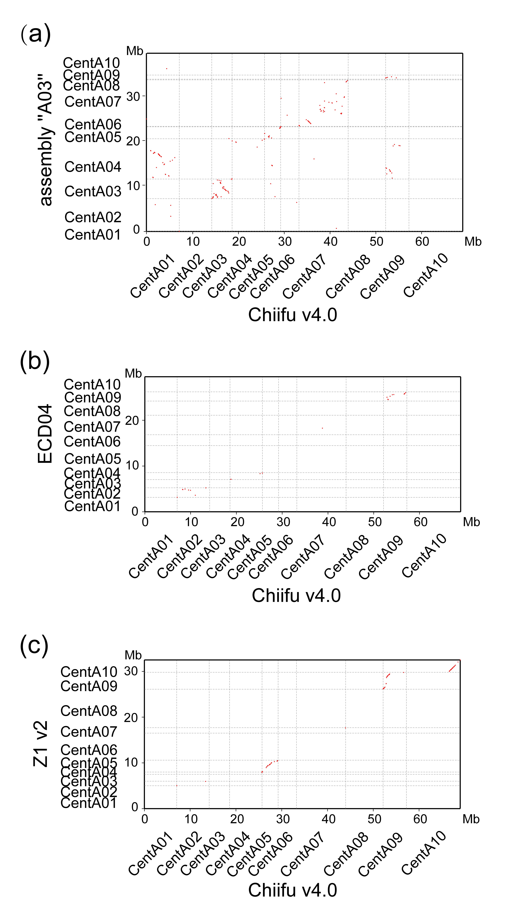


**Figure S9** **Centromere collinearity between *Brassica rapa* Chiifu v4.0 and other assemblies.** Collinear regions between Chiifu v4.0 and other assemblies are shown in red dots. Centromeres of Chiifu v4.0 are shown on the x-axis, and centromeres of other assemblies are shown on the y-axis.


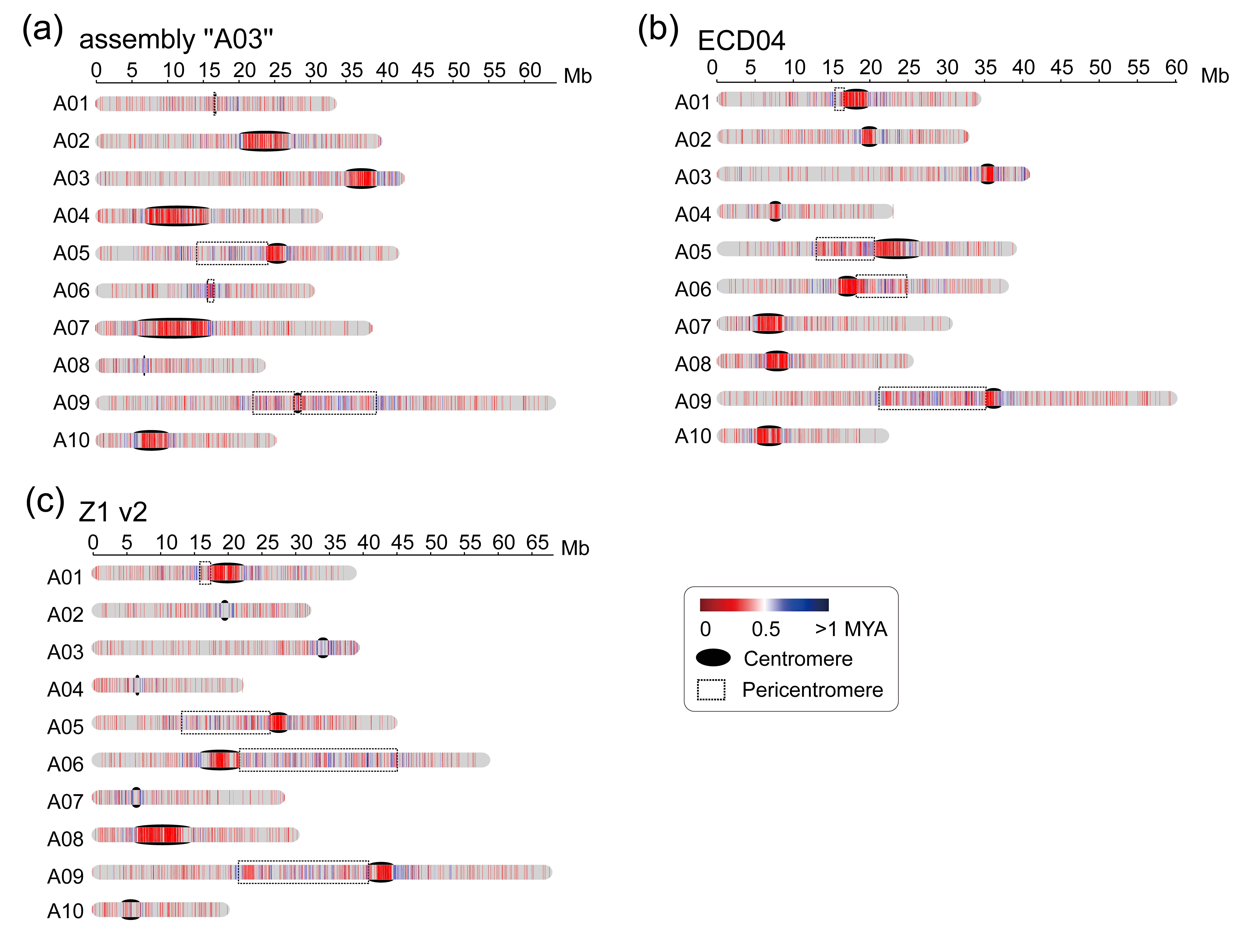


**Figure S10** **The age distribution of FL-LTR-RTs in *Brassica rapa* genome assemblies.** Black blocks indicate centromeres, and dashed blocks indicate pericentromeres.


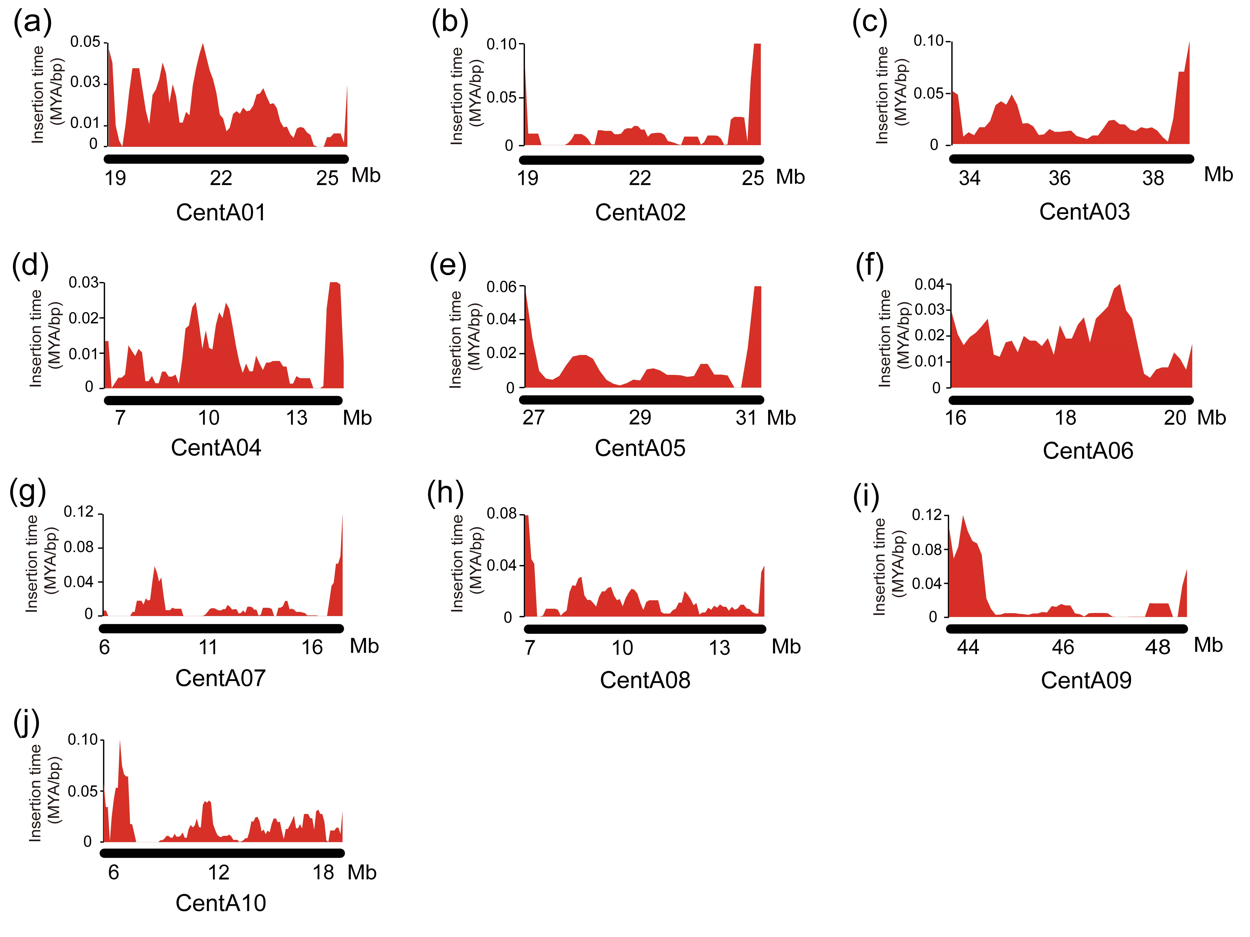


**Figure S11** **The** **density of insertion time of FL-LTR-RTs in centromeres of *Brassica* *rapa* Chiifu v4.0.** The density of insertion time was calculated using a 500 kb sliding window and 100 kb step size.


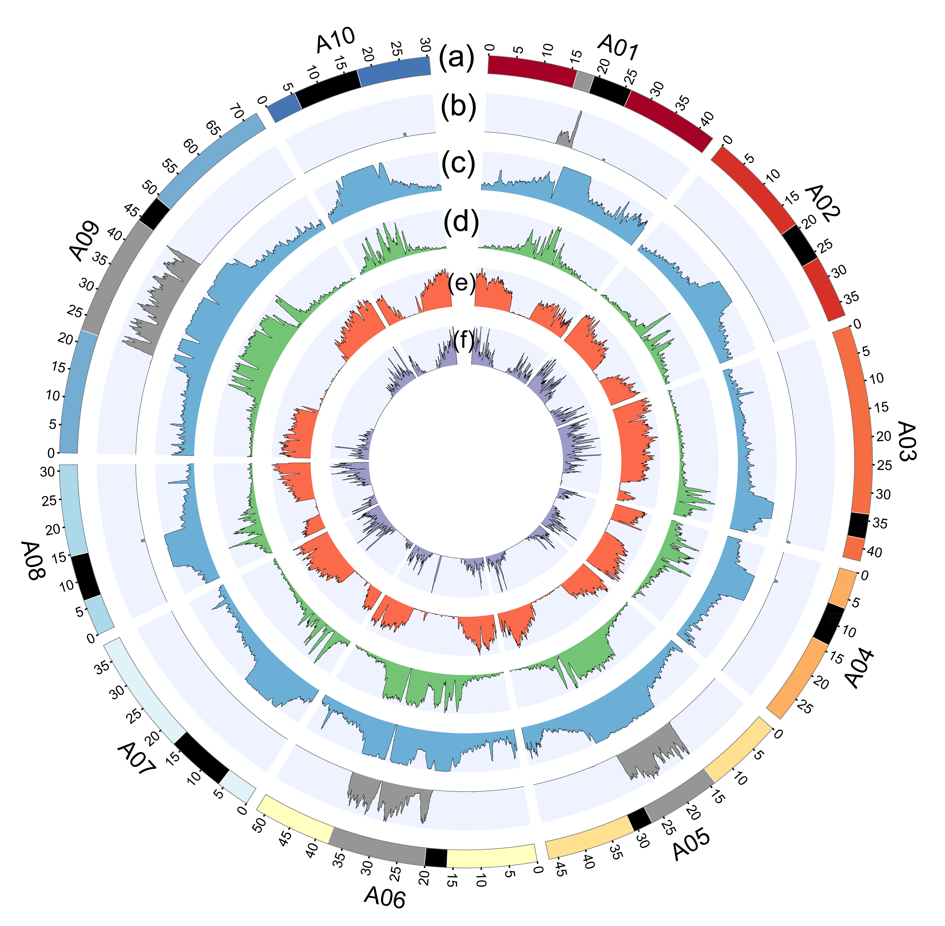


**Figure S12 Characterization of the pericentromeres in *Brassica rapa* Chiifu v4.0.** **(a)** Ten chromosomes of Chiifu v4.0. The pericentromeres are shown as grey blocks, and the centromeres are shown as black blocks. **(b)** Distribution of *PCRBr* along the chromosomes. **(c)** TEs density across the chromosomes. **(d)** LTRs density across the chromosomes. **(e)** Gene density of the chromosomes. **(f)** The expression level of genes along the chromosomes. All data were calculated by a 500 kb sliding window and 100 kb step size.


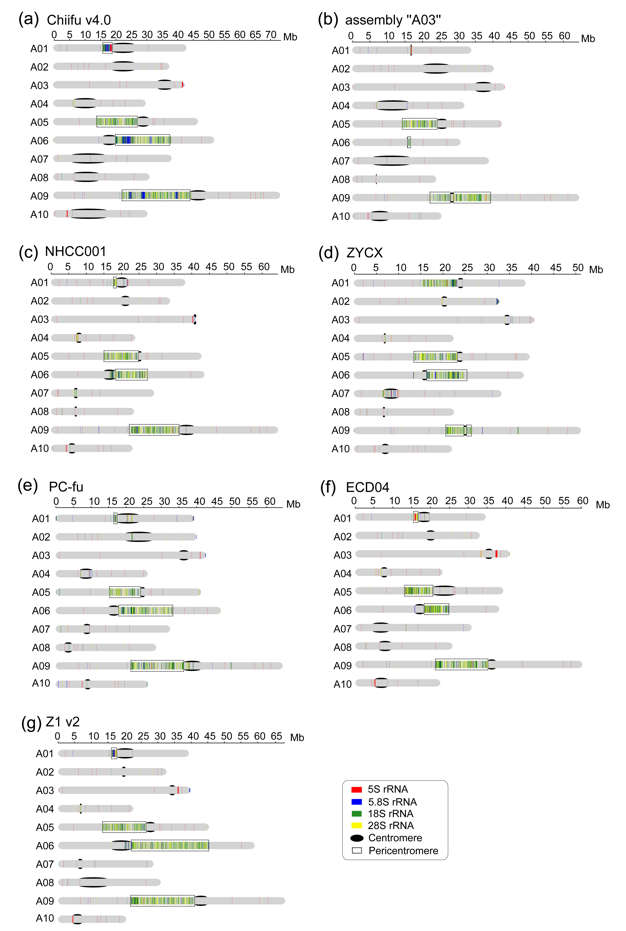


**Figure S13** **The distribution of rRNA sequences in *Brassica rapa* genome assemblies.** Black blocks indicate centromeres, and dashed blocks indicate pericentromeres.


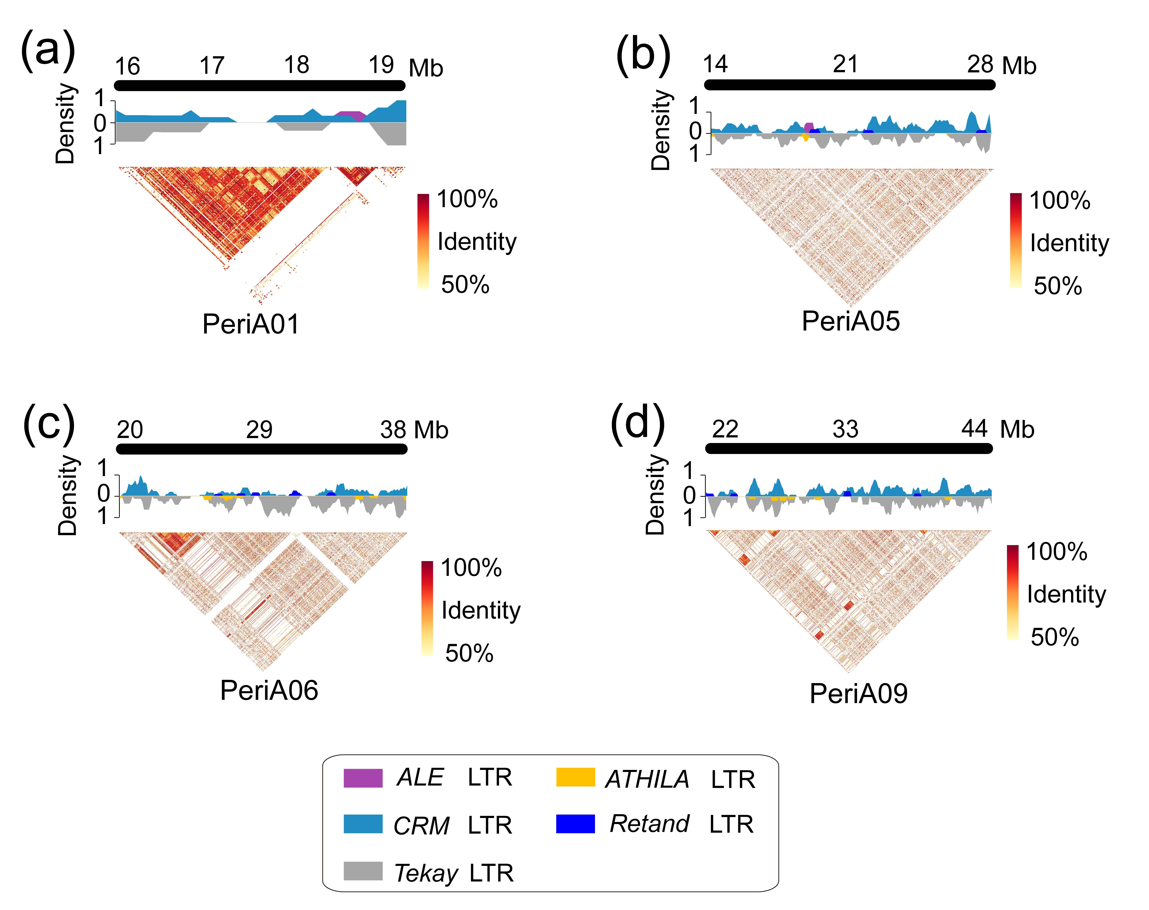


**Figure S14 Heatmap shows pairwise sequence identity between all non-overlapping 10 kb regions of pericentromeres in *Brassica* *rapa* Chiifu v4.0.**


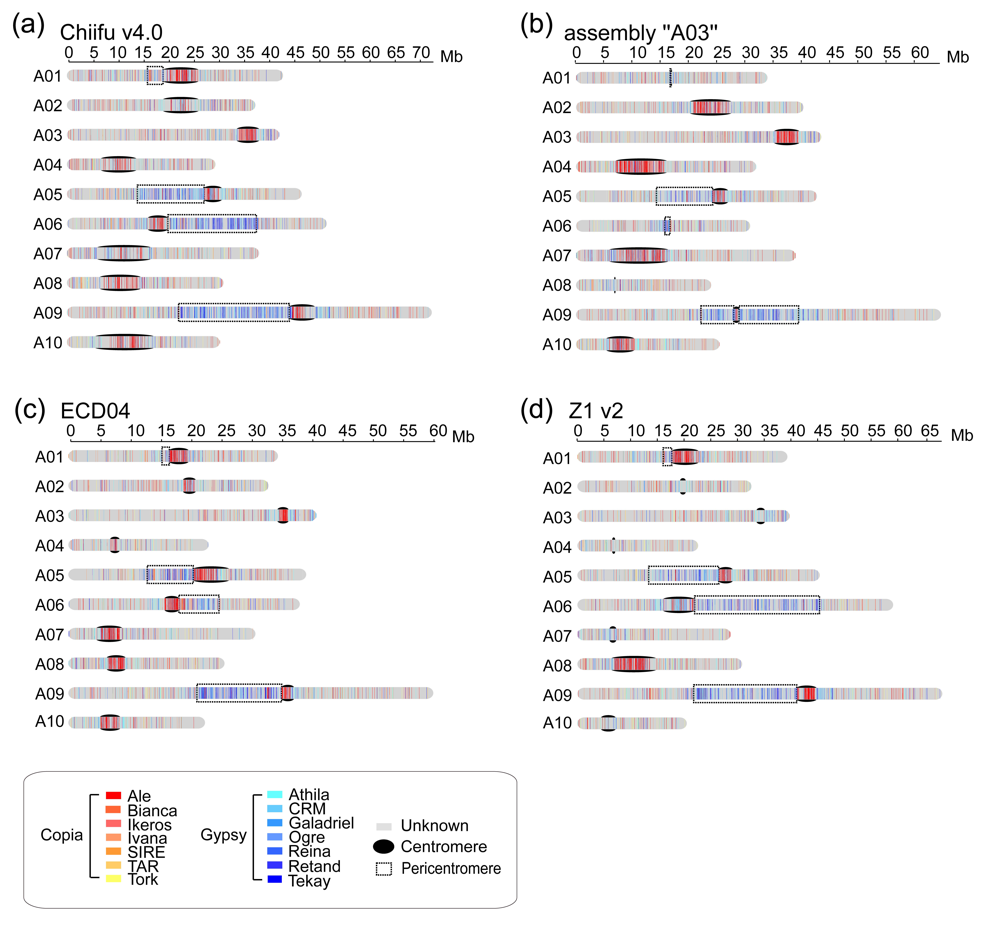


**Figure S15** **The family distribution of FL-LTR-RTs in different *Brassica rapa* genome assemblies.** Black blocks indicate centromeres, and dashed blocks indicate pericentromeres.


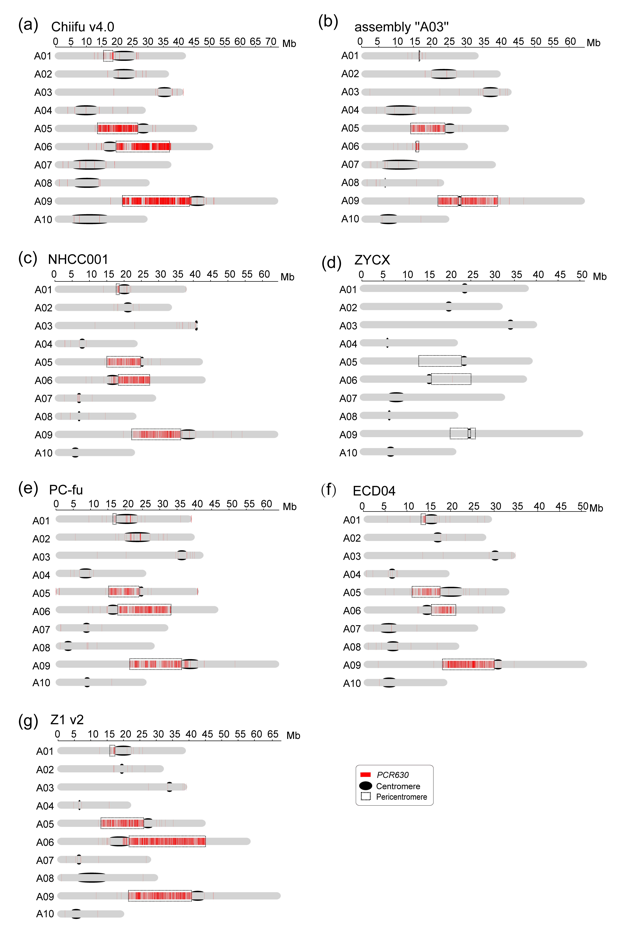


**Figure S16** **The distribution of *PCR630* in *Brassica rapa* genome assemblies.** Black blocks indicate centromeres, and dashed blocks indicate pericentromeres.


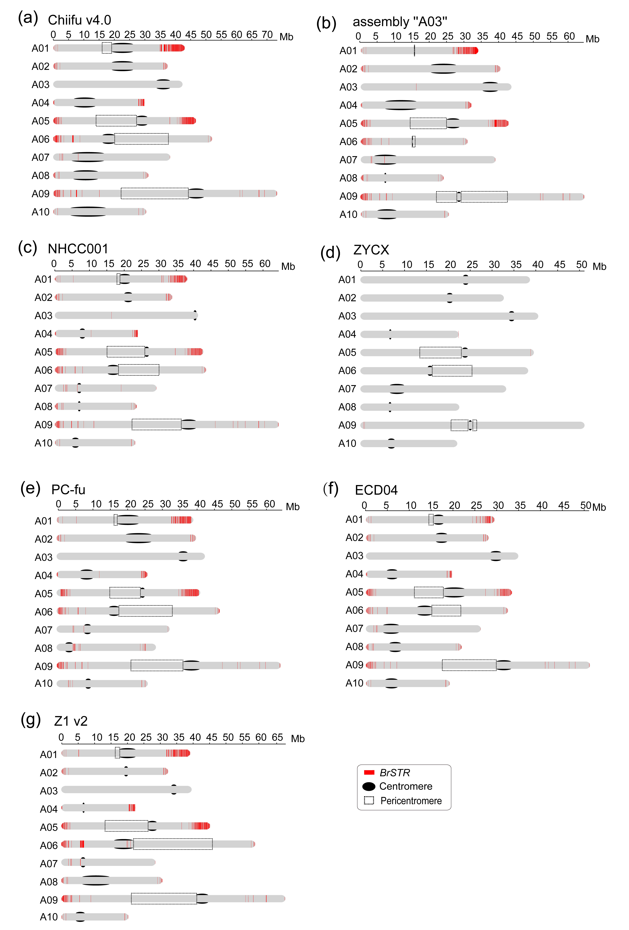


**Figure S17** **The distribution of telomere-specific repeat in *Brassica rapa* genome assemblies.** Black blocks indicate centromeres, and dashed blocks indicate pericentromeres.
